# Exercise modalities in mild cognitive impairment: a systematic review and network meta-analysis of comparative effectiveness

**DOI:** 10.3389/fnagi.2026.1888709

**Published:** 2026-07-16

**Authors:** Kaiyin Cui, Wenlang Yu, Jiabao Zhang, Bin Zheng

**Affiliations:** 1 School of Sport Science, Beijing Sport University, Beijing, China; 2 Beijing Sports Nutrition Engineering Research Center, Beijing, China; 3 Key Laboratory of the Ministry of Education of Exercise and Physical Fitness, Beijing Sport University, Beijing, China; 4 General Hospital of Northern Theater Command, Shenyang, China

**Keywords:** executive function, exercise intervention, global cognition, memory, mild cognitive impairment, network meta-analysis

## Abstract

**Background:**

Mild cognitive impairment (MCI) represents an intermediate stage between normal aging and dementia and is associated with an increased risk of progression to dementia. Exercise is a promising non-pharmacological intervention for cognitive decline; however, the comparative effectiveness of different exercise modalities across cognitive domains remains unclear.

**Objectives:**

This study aimed to compare the effects of different exercise modalities on domain-specific cognitive outcomes in patients with MCI and to identify optimal exercise prescription characteristics for the most effective intervention.

**Methods:**

This systematic review and network meta-analysis followed PRISMA guidelines and was preregistered in PROSPERO (CRD420251152983). Randomized controlled trials investigating exercise interventions in individuals with MCI were identified through database searches. Exercise modalities included aerobic exercise, resistance exercise, mind–body exercise, dance exercise, combined aerobic and resistance exercise, and multicomponent exercise, with passive and active controls as comparators. Standardized mean differences (SMDs) were calculated using change-from-baseline scores. A frequentist random-effects network meta-analysis was performed using Stata 17. Prespecified subgroup analyses examined exercise prescription characteristics for multicomponent exercise.

**Results:**

A total of 55 randomized controlled trials involving 4,603 participants were included. Network meta-analysis demonstrated that all exercise modalities were more effective than passive control in improving global cognitive function, with multicomponent exercise ranking highest (SUCRA = 83.3%). For memory-related outcomes, resistance exercise, mind–body exercise, dance exercise, and multicomponent exercise were superior to passive control, with mind–body exercise ranking highest (SUCRA = 83.9%). For executive-function-related outcomes, aerobic exercise, resistance exercise, dance exercise, combined aerobic and resistance exercise, and multicomponent exercise showed significant benefits compared with passive control, and resistance exercise ranked highest (SUCRA = 73.7%). Subgroup analyses indicated that moderate-intensity multicomponent exercise performed at least five times per week was associated with greater improvements in global cognitive function. Interventions lasting 13–24 weeks showed the largest effect estimates, although intervention period was not a statistically significant moderator.

**Conclusion:**

Different exercise modalities exhibit domain-specific cognitive benefits in individuals with MCI. Multicomponent exercise appears most effective for improving global cognitive function, mind–body exercise may preferentially benefit memory-related outcomes, and resistance exercise may provide greater benefits for executive-function-related outcomes. These findings support individualized selection of exercise modalities for individuals with MCI.

**Systematic review registration:**

https://www.crd.york.ac.uk/PROSPERO/view/CRD420251152983, Identifier: CRD420251152983.

## 1 Introduction

Mild cognitive impairment (MCI) is characterized by mild deficits in one or more cognitive domains—most commonly memory, language, attention, and executive function—while basic activities of daily living remain largely preserved. It is widely regarded as a transitional stage between normal aging and dementia (Anderson, 2019). The prevalence of MCI among adults aged ≥65 years is estimated at 10%–15% (Anderson, 2019). However, limited awareness and the lack of effective screening and intervention systems often delay identification and management, resulting in missed opportunities for early intervention (Yu et al., 2024; Li et al., 2022; Zhao et al., 2022; Xue et al., 2023). Individuals with MCI have a substantially higher risk of progressing to dementia than cognitively normal older adults, creating a considerable burden on patients, caregivers, and healthcare systems (Yu et al., 2024; Qiu et al., 2025).

In the absence of effective disease-modifying therapies, exercise has emerged as a promising non-pharmacological strategy for delaying cognitive decline. Evidence suggests that aerobic exercise, resistance training, mind–body exercise, and multicomponent exercise can improve cognitive function in individuals with MCI. Resistance training has been shown to enhance memory, processing speed, and executive-related abilities (Yoon et al., 2018), whereas aerobic and strength training may improve global cognitive performance (Silva et al., 2025). Proposed mechanisms include improvements in cardiorespiratory fitness, metabolic regulation, neurotrophic signaling, cerebral perfusion, and neuroplasticity (Nuzum et al., 2020; Sánchez-Alcalá et al., 2025; Tarumi et al., 2022; Bao et al., 2021). Traditional mind–body exercise and multicomponent programs have also demonstrated benefits for cognition, physical function, and overall well-being (Pieruccini-Faria et al., 2025; Zhang et al., 2024; Young et al., 2024; Khattak et al., 2024; Lin et al., 2025; Xu et al., 2023; Wu et al., 2023).

Several systematic reviews and network meta-analyses have compared the relative efficacy of exercise modalities for cognitive impairment (Huang et al., 2022; Cai et al., 2023; Chen et al., 2025). Huang et al. (Huang et al., 2022) reported differential effects across cognitive domains in a network meta-analysis including both MCI and dementia populations, suggesting that exercise prescriptions may need to be tailored to specific cognitive outcomes. However, combining MCI and dementia populations may obscure disease-stage–specific responses to exercise interventions. Furthermore, most available studies compare a single exercise modality with a control condition, limiting direct comparisons across exercise modalities. Several exercise modalities and recently published randomized controlled trials were unavailable at the time of previous reviews, and exercise prescription characteristics associated with the most effective interventions were not systematically examined. Consequently, uncertainty remains regarding the optimal exercise modality for specific cognitive domains, as well as the optimal combination of exercise intensity, frequency, session duration, and intervention period for individuals with MCI.

Therefore, an updated network meta-analysis focusing exclusively on individuals with MCI is warranted. Compared with previous reviews, the present study (1) included only participants with MCI, (2) incorporated evidence published through September 2025, (3) evaluated additional exercise modalities, and (4) explored exercise prescription characteristics associated with the most effective intervention. Accordingly, we conducted a systematic review and network meta-analysis to compare the effects of different exercise modalities on global cognition, memory, and executive function in individuals with MCI and to identify exercise prescription characteristics associated with optimal cognitive outcomes.

## 2 Methods

This systematic review was conducted in accordance with the PRISMA guidelines and was prospectively registered in PROSPERO (CRD420251152983). The review methodology followed the recommendations of the Cochrane Handbook for Systematic Reviews of Interventions (Page et al., 2021).

### 2.1 Data sources and searches

A comprehensive literature search was conducted using a combination of Medical Subject Headings (MeSH) and free-text terms. The target population was MCI, including MCI - amnestic type and MCI - non-amnestic type. Exercise-related interventions included physical activity, exercise, bodily activity, aerobic exercise, moderate-intensity continuous training (MICT), high-intensity interval training (HIIT), high-intensity circuit training (HICT), resistance training, strength training, flexibility training, balance training, home-based exercise, rehabilitation exercise, aquatic rehabilitation, Tai Chi, dance, and martial arts. Outcomes of interest were changes in cognitive function (e.g., cognition and cognitive ability). Eligible study designs were randomized controlled trials (RCTs), including randomized and randomised controlled trials.

The following electronic databases were systematically searched: PubMed, EBSCO, the Cochrane Library, Web of Science, and Embase. The search period covered each database from inception to 19 September 2025. In addition, reference lists of included studies were manually screened to identify potentially relevant articles that may have been missed. Studies examining the effects of exercise on neurodegenerative diseases were also considered to ensure comprehensive coverage.

The complete PubMed search strategy is provided in Appendix 1A. The same search concepts and Boolean operators were applied across all databases, with minor adaptations made where necessary to accommodate differences in indexing systems and search interfaces.

### 2.2 Study selection

Two reviewers independently screened the literature and extracted data in accordance with the predefined eligibility criteria. Discrepancies were resolved through discussion; if consensus could not be reached, a third reviewer was consulted. Data extracted from each included study comprised the first author, publication year, participant characteristics (age and sex), intervention characteristics (exercise modality, frequency, and implementation), and outcome measures. Inter-rater agreement for study selection was quantified using Cohen’s kappa coefficient calculated with SPSS software.

#### 2.2.1 Inclusion criteria

Studies were eligible for inclusion if they met the following criteria. First, studies had to adopt a randomized controlled trial (RCT) design. Participants were required to be adults diagnosed with mild cognitive impairment (MCI) according to the Petersen criteria (2001), the National Institute on Aging–Alzheimer’s Association (NIA-AA) criteria (2011), or other widely accepted diagnostic frameworks consistent with established definitions of MCI and explicitly described in the original studies. No restriction on minimum age was imposed at the eligibility stage; however, all included trials enrolled middle-aged or older adults, with reported mean ages ranging from approximately 60 to 85 years. Studies enrolling participants with other specific neurological or psychiatric diseases known to cause cognitive impairment were excluded.

The intervention group had to receive any form of structured exercise training. Eligible interventions were required to involve repeated exercise sessions delivered over a period of at least 1 week. Control conditions were required to involve no structured exercise training and could include no intervention (blank control), usual care (e.g., routine health monitoring without additional exercise), health education only (provision of health-related information without exercise guidance), sham exercise (e.g., low-intensity, non–goal-directed physical activity without expected training effects), or other exercise modalities for head-to-head comparisons between different exercise types. In addition, eligible studies were required to report at least one cognitive outcome of interest (e.g., MMSE, MoCA, GPCOG, MDRS, TICS-M, or ADAS-Cog–Executive Function) with sufficient quantitative data for effect-size estimation. To ensure accurate data extraction and minimize potential translation bias, only full-text articles published in English were included.

#### 2.2.2 Exclusion criteria

Studies were excluded if they met any of the following criteria: (1) reviews, systematic reviews, meta-analyses, study protocols, conference abstracts/proceedings, or other non–full-text publications; (2) studies involving populations outside the scope of the present review, including individuals diagnosed with Alzheimer’s disease, Parkinson’s disease, Huntington’s disease, epilepsy, multiple sclerosis, diabetes, or major psychiatric disorders (e.g., schizophrenia); (3) studies in which the intervention was not exercise-based; (4) studies evaluating only acute exercise effects or single-session exercise interventions (e.g., a single 30-min exercise session); (5) non-randomized study designs, including non-randomized controlled trials, observational studies, case reports, and case series; (6) studies without accessible full texts or studies from which valid outcome data could not be extracted despite attempts to contact the corresponding authors; (7) duplicate publications of the same trial, in which case the most complete report was retained; and (8) animal studies, *in vitro* experiments, or other non-human research.

### 2.3 Data extraction and quality assessment

Data extraction was performed independently by two reviewers using a standardized data extraction form. Any discrepancies were resolved through discussion or, when necessary, by consultation with a third reviewer. The risk of bias of individual randomized controlled trials was assessed independently by two reviewers using the Cochrane Risk of Bias tool, version 1 (RoB 1). Bias was evaluated across the standard domains specified in RoB 1, and each study was classified as having low risk, some concerns, or high risk of bias. The certainty of evidence for each network comparison was evaluated using the Grading of Recommendations Assessment, Development and Evaluation (GRADE) approach (Salanti et al., 2014).

Exercise interventions were categorized according to their primary training characteristics and intended physiological or cognitive targets (Chodzko-Zajko et al., 2009; Garber et al., 2011; Bull et al., 2020). Interventions with predominantly cardiorespiratory training components, such as walking, cycling, treadmill exercise, and aerobic gymnastics, were classified as aerobic exercise (AE) (Chodzko-Zajko et al., 2009; Garber et al., 2011; Bull et al., 2020). Interventions primarily designed to improve muscular strength, endurance, or power through external resistance, including free-weight training, machine-based resistance training, and elastic-band exercise, were classified as resistance exercise (RE) (Chodzko-Zajko et al., 2009; Garber et al., 2011; Bull et al., 2020). Mind–body exercise (MBE) included interventions integrating physical movement with breathing regulation, cognitive focus, and meditative elements, such as Tai Chi, Baduanjin, and Qigong (Wayne and Kaptchuk, 2008). Dance-based interventions involving coordinated movement patterns performed to music were classified as dance exercise (DE) (Huang et al., 2022). Programs incorporating both aerobic and resistance training components within the same intervention were classified as combined aerobic and resistance exercise (ARE) (Huang et al., 2022). Interventions combining three or more exercise components, such as aerobic, resistance, balance, flexibility, coordination, or cognitive–motor dual-task training, were classified as multicomponent exercise (MCE) (Huang et al., 2022; Cadore et al., 2013).

### 2.4 Data synthesis and statistical analysis

Continuous outcomes were summarized as means and standard deviations (SDs). Effect sizes were calculated as standardized mean differences (SMDs) based on change-from-baseline scores, with outcome directions aligned such that positive values indicated improvements in cognitive performance. For studies reporting sufficient variance information (e.g., confidence intervals or standard errors), SDs were directly derived using standard statistical conversions. When SDs of change scores were unavailable, they were imputed from baseline and post-intervention SDs using the Cochrane-recommended equation (Equation 1), assuming a correlation coefficient of r = 0.5 between baseline and post-intervention measurements:


$$SD_{\Delta }=\sqrt{SD_{\text{baseline}}^{2}+SD_{\text{post}}^{2}-2r\cdot SD_{\text{baseline}}\cdot SD_{\text{post}}}$$ (1)

Where *r* represents the assumed correlation coefficient between baseline and post-intervention measurements. Sensitivity analyses using alternative correlation coefficients (*r* = 0.3 and *r* = 0.7) were conducted for the global cognition outcome. However, such analyses were not performed for memory-related and executive-function-related outcomes because a substantial proportion of studies directly reported change-score SDs or provided sufficient statistical information for variance derivation, resulting in heterogeneous sources of variance estimation across studies.

For studies including multiple intervention arms within the same exercise modality, relevant arms were combined into a single node to avoid double counting of participants in the network meta-analysis.

Cognitive outcomes were synthesized by domain. For global cognitive function and cognition-related memory function, each study contributed a single representative outcome to the analysis to avoid unit-of-analysis errors arising from multiple correlated measures within the same trial. When multiple eligible outcomes were reported, one outcome was selected *a priori* according to a predefined hierarchy: (1) the trial-specified primary cognitive or memory outcome; if unavailable, (2) delayed episodic memory measures (delayed recall or recognition); otherwise, (3) the most widely used and comparable test across studies. All selected outcomes were analyzed using change-score SMDs.

For cognition-related executive function, given the heterogeneity of executive test batteries across trials, executive outcomes were synthesized at the study level. Within each study, all eligible executive-related measures were directionally aligned so that higher values consistently reflected better performance. Individual change scores were then standardized by dividing the post–pre difference by the corresponding baseline standard deviation (Equation 2) (Song et al., 2013):


$$z_{i}=\frac{\left(\mathrm{Pos}t_{i}-\mathrm{Pr}e_{i}\right)}{SD_{\text{baseline},i}}$$ (2)

These standardized change scores were subsequently aggregated into a single composite executive function score within each study by calculating their arithmetic mean (Equation 3) (Song et al., 2013):


$$z_{\text{composite}}=(\frac{1}{k})\sum_{i=1}^{k}z_{i}$$ (3)

Where *k* denotes the number of executive-related outcomes available in the study. The resulting composite change *z* scores and their corresponding standard deviations were subsequently converted into SMDs and included in the meta-analysis.

The specific outcome measures contributing to the global cognition and memory analyses, as well as the executive-function outcomes aggregated within each study, are reported in Appendix 1B.

Random-effects network meta-analyses were conducted within a frequentist framework using Stata (version 17). Between-study heterogeneity was quantified using the heterogeneity variance (*τ*^2^). Network geometry and the contribution of direct and indirect evidence were examined using network plots and contribution plots. Consistency was assessed using both global (design-by-treatment interaction model) and local (node-splitting) approaches. In the absence of important inconsistency, results from the consistency model were reported.

Treatment rankings were estimated using cumulative ranking probabilities and summarized by the surface under the cumulative ranking curve (SUCRA). Potential small-study effects and publication bias were explored using comparison-adjusted funnel plots.

The certainty of evidence for each network estimate was assessed using the Grading of Recommendations Assessment, Development and Evaluation (GRADE) framework adapted for network meta-analysis, informed by the Confidence in Network Meta-Analysis (CINeMA) approach. The assessment considered within-study risk of bias, inconsistency, indirectness, imprecision, and publication bias.

## 3 Results

### 3.1 Study search and selection

**Figure 1:**
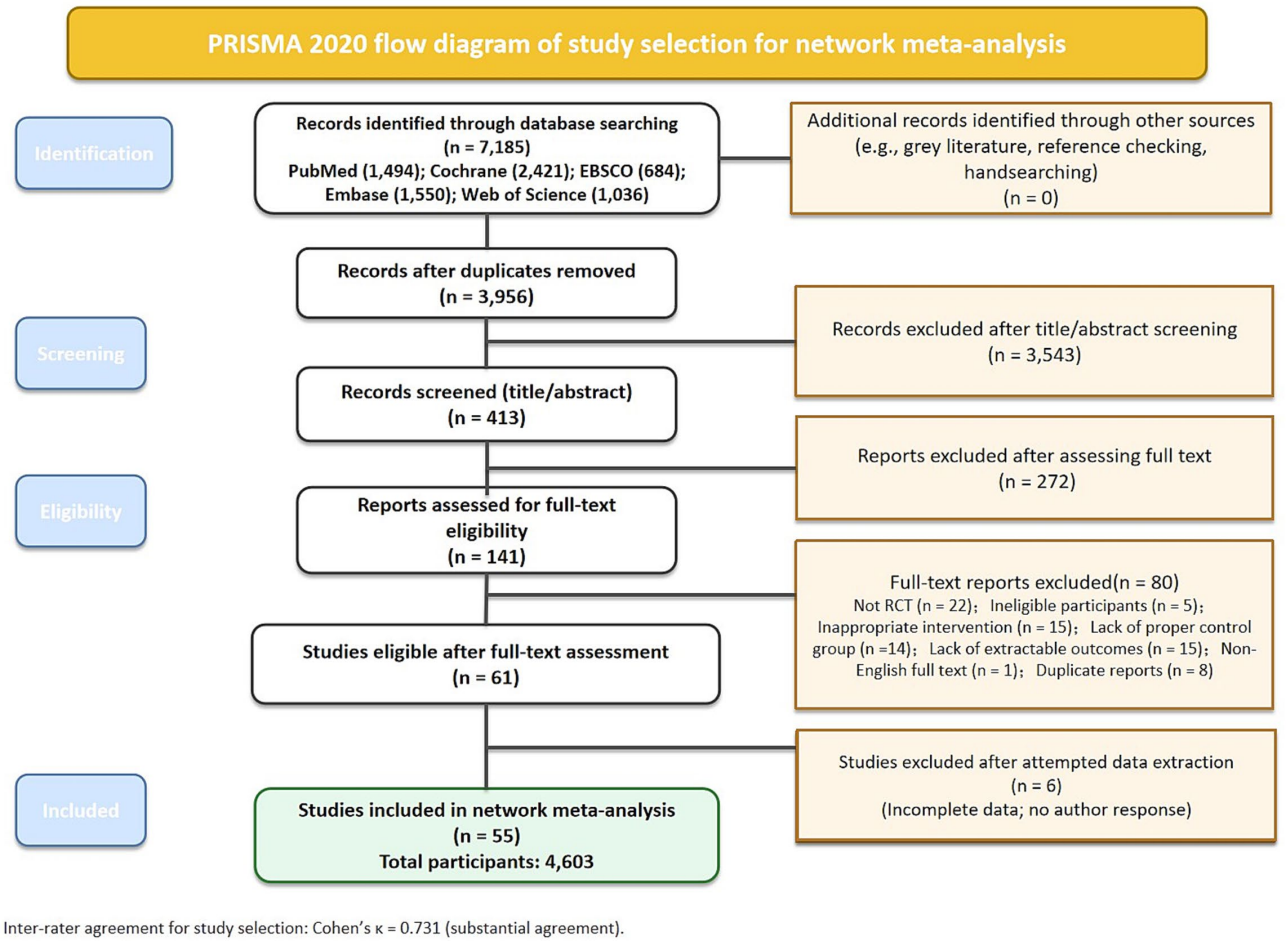
PRISMA 2020 flow diagram of study selection for network meta-analysis.

A total of 7,185 records were identified through database searching, including 1,494 from PubMed, 2,421 from the Cochrane Library, 684 from EBSCO, 1550 from Embase, and 1,036 from Web of Science. Following the PRISMA flow, 3,956 records remained after removal of duplicates. After comprehensive screening of titles and abstracts, 413 articles were retained for full-text assessment. Based on the predefined inclusion and exclusion criteria, 61 studies were eligible for further evaluation. Outcome data were subsequently extracted from these studies; 6 studies were excluded due to incomplete data and lack of response from the corresponding authors. Ultimately, 55 studies involving a total of 4,603 participants were included in the network meta-analysis. The detailed search and study selection process is presented in Figure 1.

Inter-rater agreement between the two reviewers during the study selection process was substantial, with a Cohen’s kappa coefficient of 0.731 (*κ* > 0.60), indicating good consistency.

### 3.2 Characteristics of included studies

The main characteristics of the 55 included randomized controlled trials are summarized in Table 1. All studies enrolled participants diagnosed with MCI and compared one or more exercise interventions with either active or passive control conditions. Detailed intervention characteristics and control conditions for all included studies are summarized in Appendix 1B.

**Table 1:** Characteristics of included studies.

Study	Country	Sample size (IG/CG)	MCI diagnostic criteria	Age	Intervention (Exercise type)	Control intervention	Outcome measures
Bademli et al. (2019)	Turkey	30/30	Meets MCI criteria	71.5 ± 7.8	MCE	PC	SMMSE
Bisbe et al. (2020)	Spain	18/18	Petersen’s criteria	75.0 ± 5.5	DE, MCE	—	MMSE; RBANS VDR; TMT-A; TMT-B; LVFT; CVFT
Chang et al. (2021)	China	72/64	Meets MCI criteria	76.29 ± 3.60	DE	PC	MoCA
De Sa et al. (2024)	Brazil	9/9/9	Meets MCI criteria	71.3 ± 6.6	ARE, MCE	AC	MMSE
Doi et al. (2017)	Japan	67/67	Petersen’s criteria	76.0 ± 4.5	DE	PC	MMSE; Story Memory; TMT-A; TMT-B
Fiatarone Singh et al. (2014)	Australia	25/25	Petersen’s criteria	70.1 ± 6.7	RE	AC	ADAS-Cog; BVRT; WAIS-III Similarities; WAIS-III Matrices; LVFT; CVFT
Fonte et al. (2019)	United States	7/9	NIA-AA core clinical criteria	77 ± 4.4	ARE	PC	MMSE; RBMT; TMT-A; TMT-B; FAB
Hwang H. F. et al. (2023)	China (Taiwan)	41/56	Petersen’s criteria	≥65	MBE	AC	MDRS
Jiang et al. (2019)	China	22/22	Meets MCI diagnosis	60–70	MCE	PC	MoCA
Khanthong et al. (2021)	Thailand	35/36	Meets amnestic MCI	60.9 ± 6.6	MBE	PC	MoCA; TMT-A; TMT-B
Khattak et al. (2024)	Pakistan	26/25	Meets MCI criteria	62.74 ± 7.4	AE, MCE	PC	MoCA
Kohanpour et al. (2017)	Iran	10/10	Meets MCI criteria	68.13 ± 3.71	AE	PC	MMSE
Krootnark et al. (2024)	Thailand	30/30	Meets MCI criteria	69.00 ± 5.03	AE, RE	PC	MoCA; DST-B; TMT-B; SCWT III
Labott et al. (2025)	Germany	24/22	Winblad & Petersen criteria	69.9 ± 6.2	DE	PC	MMSE; TMT-A; TMT-B
Lam et al. (2011)	China (Hong Kong)	171/218	Meets MCI criteria	77.8 ± 6.5	MBE	AC	CMMSE; Delayed Recall; CVFT; TMT-A (Chinese version); TMT-B (Chinese version); DST-B
Lam et al. (2014)	China (Hong Kong)	171/218	Petersen’s criteria	77.8 ± 6.5	MBE	AC	MMSE; Delayed recall
Lam et al. (2015)	China (Hong Kong)	147/131	Meets MCI criteria	75.4 ± 6.5	MCE	AC	CMMSE; Delayed Recall; CVFT; TMT-A (Chinese version); TMT-B (Chinese version); DST-B
Langoni et al. (2019)	Brazil	26/26	Meets MCI criteria	72.3 ± 7.9	ARE	PC	MMSE
Lazarou et al. (2017)	Greece	66/63	Petersen’s criteria	66.9 ± 10.1	DE	PC	MoCA; RBMT 2
Li et al. (2021)	China	42/42	Petersen’s criteria	60–79	MCE	PC	MoCA
Li et al. (2023)	United States	107/106	Meets criteria for MCI or self-reported memory concern	76.0 ± 5.4	MBE	AC	MoCA
Lin et al. (2024)	China	50/50	Petersen’s criteria	68.1 ± 4.9	MBE	PC	MoCA; WMS-MQ
Lin et al. (2025)	China	18/18/18	Meets criteria of Chinese Alzheimer’s Disease Association	84.46 ± 5.96	AE, MBE	PC	MoCA; AVLT-Delayed
Liu et al. (2021)	China	17/20/20	Petersen’s criteria	65.5 ± 4.5	AE, MBE	PC	MoCA
Liu et al. (2022)	China (Taiwan)	18/18	Petersen’s criteria	73.7 ± 6.3	MBE	PC	MoCA; TMT-A; TMT-B; Delta-TMT
Lü et al. (2016)	China	22/23	Petersen’s criteria	69.7 ± 4.7	MCE	PC	ADAS-Cog
Mollinedo Cardalda et al. (2019)	Spain	25/23/29	Blesa & Lobo et al. (2001; 1999) criteria: clinical validity of Spanish MMSE	84.76 ± 7.91	RE, DE	PC	MMSE
Montero-Odasso et al. (2023)	Canada	35/34	Petersen/NIA-AA criteria	73.1 ± 6.6	ARE	AC	MoCA; Delayed Recall; TMT-A; TMT-B; DSST; DST-F; DST-B
Nascimento et al. (2015)	Brazil	24/21	Petersen’s criteria	67.7 ± 5.9	MCE	PC	MoCA
Pieruccini-Faria et al. (2025)	Canada	34/33	Petersen’s criteria	73.08 ± 6.59	ARE	AC	MoCA
Qi et al. (2019)	China	16/16	NIA-AA criteria	69.9 ± 7.2	DE	PC	MoCA; WMS-LMII
Rivas-Campo et al. (2023)	Colombia	64/68	screened by MMSE	77.2 ± 7.6	MCE	PC	MoCA; TMT-A; TMT-B; VFT; DSST; d2-CON
Rojasavastera et al. (2020)	Thailand	11/11	NIA-AA core clinical criteria	66.95 ± 4.43	AE	PC	MoCA
Silva et al. (2025)	Multicenter (Portugal, Serbia, Italy, Poland)	97/42/29	MoCA score ≤26	72.9 ± 6.0	AE, RE	PC	MoCA
Song and Yu (2019)	China	60/60	Meets MCI diagnosis	75.78 ± 6.28	AE	PC	MoCA
Song et al. (2023)	China	45/44	MoCA score 19–26	75.97 ± 6.31	DE	PC	MoCA
Suzuki et al. (2012)	Japan	25/25	Petersen’s criteria	76.05 ± 7.16	MCE	PC	MMSE; WMS-LMII; DSC; LVFT; CVFT; SCWTI; SCWT II
Suzuki et al. (2013)	Japan	47/45	Petersen’s criteria	75.3 ± 6.8	MCE	PC	MMSE; WMS-LMII
Tao et al. (2019)	China	17/20/20	Petersen’s criteria	65.55 ± 4.40	AE, MBE	PC	MoCA
Tomoto et al. (2021)	United States	22/30	Petersen’s criteria	65.5 ± 6.6	AE	AC	MMSE
Uysal et al. (2023)	Turkey	12/12	Diagnosed with MCI by clinical psychiatrist	73.7 ± 6.2	RE, ARE	—	MMSE
Varela et al. (2012)	Spain	53/15	criteria of Spanish Society of Geriatrics and Gerontology	78.3 ± 9.5	AE	AC	MMSE
Wang et al. (2020)	China	58/58	Petersen’s criteria	68.31 ± 5.19	MCE	PC	MoCA
Wang et al. (2024)	China	30/30	clinical diagnosis of MCI	60 ~ 69	DE	PC	MoCA; SCWT III; N-back Task; more-odd shifting task
Wei and Ji (2014)	China	30/30	DSM-IV and Shanghai Mental Health Center criteria	66.0 ± 5.1	MCE	PC	MMSE
Wu H. et al. (2025)	China	16/16	NIA-AA criteria	69.85 ± 7.15	DE	PC	MoCA; WMS-LMII
Wu T. et al. (2025)	China	34/34	Meets MCI criteria	72.8 ± 6.1	RE	AC	MoCA; AVLT-Delayed
Xia et al. (2022)	China	45/45/45	Petersen’s criteria	65.88 ± 4.45	AE, MBE	PC	MoCA
Yang et al. (2022)	South Korea	33/33	Meets MCI diagnosis	70.8 ± 5.4	ARE	PC	MMSE; TMT-A; DSST
Yoon et al. (2017)	South Korea	23/7	Petersen’s criteria	75.8 ± 3.7	RE	AC	MMSE
Yu A. P. et al. (2022)	China (Hong Kong)	10/12/12	Meets MCI criteria	67.4 ± 6.4	MBE, MCE	—	MoCA-HK; Delayed Recall; TMT-B; Delta TMT; TMT B/A ratio
Yu D. J. et al. (2022)	China	30/7	Mayo Clinic criteria	63.5 ± 5.1	AE	AC	HK-MoCA
Zhang et al. (2023)	China	14/14	Meets MCI diagnosis	67.55 ± 6.22	AE	PC	MoCA
Zheng et al. (2021)	China	20/20/20	Petersen’s criteria	65.51 ± 4.38	AE, MBE	PC	MoCA; WMS-MQ
Zhou (2025)	China	22/22	Meets MCI criteria	65.8 ± 5.5	MBE	PC	MoCA; WMS-LM II; SCWT; TMT-A; CDT

Sample size (IG/CG): Sample size (Intervention/ Control intervention). Numbers indicate intervention types: passive control (PC); active control (AC); aerobic exercise (AE); resistance exercise (RE); mind–body exercise (MBE); dance exercise (DE); combined aerobic and resistance exercise (ARE); and multicomponent exercise (MCE).

Across the included trials, six exercise modalities were investigated for their effects on cognitive outcomes. Specifically, 15 studies evaluated AE, 7 assessed RE, 14 examined MBE, 10 investigated DE, 7 evaluated combined ARE, and 15 assessed MCE. Several trials included more than one exercise intervention arm.

Control conditions were categorized as passive control (PC), including no intervention or usual care, and active control (AC), such as health education, stretching, or low-intensity activities without a targeted training effect. The duration, frequency, and intensity of exercise interventions varied across studies, reflecting heterogeneity in exercise prescriptions.

Overall, the included trials provided a broad representation of exercise modalities and control conditions, allowing for comprehensive comparisons of their relative effects on cognitive outcomes in individuals with MCI.

### 3.3 Risk of bias assessment and certainty of evidence

**Figure 2:**
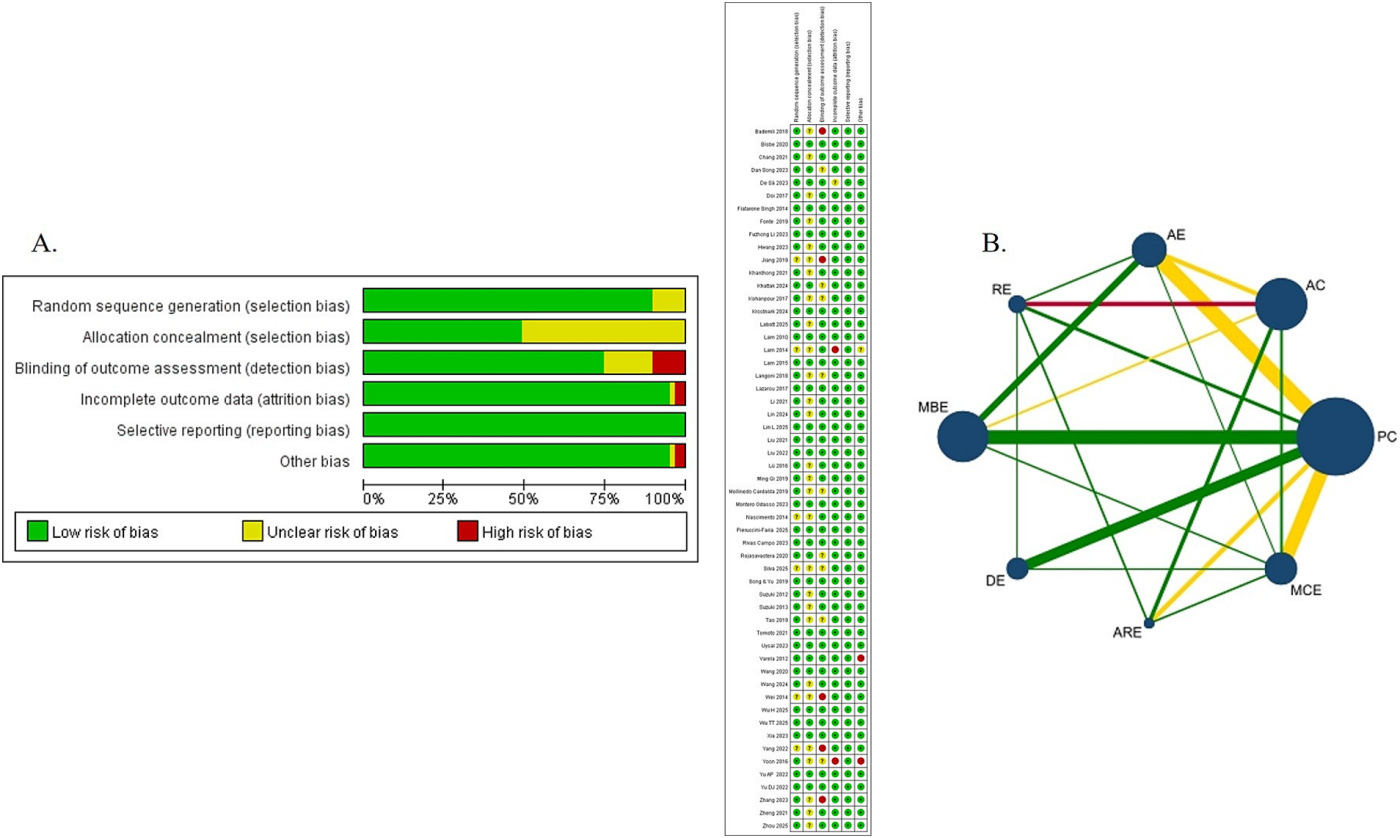
Risk-of-bias assessment and network geometry of included studies. **(A)** Risk-of-bias summary across included studies; **(B)** Network geometry weighted by risk of bias for global cognitive outcomes.

The risk-of-bias assessments of included studies are summarized in Figure 2A. For random sequence generation, 89.8% of studies were judged as low risk of bias, 10.2% as unclear risk, and none as high risk. For allocation concealment, 49.2% of studies were rated as low risk and 50.8% as unclear risk. Regarding blinding of outcome assessment, 74.6% of studies were assessed as low risk, 15.3% as unclear risk, and 10.2% as high risk. Incomplete outcome data were judged as low risk in 94.9% of studies, while 1.7% and 3.4% were assessed as unclear and high risk, respectively. Selective outcome reporting was rated as low risk across all included studies. Other sources of bias were uncommon, with 94.9% of studies assessed as low risk, 1.7% as unclear risk, and 3.4% as high risk. Additional supplementary figures related to risk-of-bias assessments and network analyses for global cognitive function are provided in Appendix 2.

The certainty of evidence for network estimates was evaluated using the CINeMA framework across six domains, including within-study bias, reporting bias, indirectness, imprecision, heterogeneity, and incoherence. The network geometry weighted by risk of bias is presented in Figure 2B. Overall, most direct comparisons between exercise interventions and passive control were rated as high confidence. In contrast, several comparisons involving active control or indirect evidence were downgraded because of imprecision, indirectness, or inconsistency. Specifically, comparisons such as AE vs. RE, RE vs. MCE, and ARE vs. MCE were downgraded because of local inconsistency identified in node-splitting analyses, whereas several indirect comparisons (e.g., RE vs. MBE, MBE vs. DE, and DE vs. ARE) were downgraded because of concerns regarding indirectness and transitivity assumptions. Imprecision due to wide confidence intervals crossing the line of no effect was the most common reason for downgrading. Overall, the confidence ratings for most network estimates ranged from moderate to high, while the ranking of treatments was judged as high confidence. Detailed CINeMA confidence ratings and reasons for downgrading are presented in Table 2.

**Table 2:** CINeMA assessment of confidence in network meta-analysis estimates for global cognitive outcomes in patients with mild cognitive impairment.

Comparison	Type of evidence	Confidence rating	Reasons for downgrading
AE vs. PC	Mixed	High	None
RE vs. PC	Mixed	High	None
MBE vs. PC	Mixed	High	None
DE vs. PC	Mixed	High	None
ARE vs. PC	Mixed	High	None
MCE vs. PC	Mixed	High	None
AE vs. AC	Mixed	Moderate	Imprecision^4^
RE vs. AC	Mixed	Moderate	Imprecision^4^
MBE vs. AC	Mixed	Moderate	Imprecision^4^
DE vs. AC	Indirect	Moderate	Imprecision^4^
ARE vs. AC	Mixed	High	None
MCE vs. AC	Mixed	High	None
AE vs. RE	Mixed	Low	Inconsistency^1^; Imprecision^4^
AE vs. MBE	Mixed	Moderate	Imprecision^4^
AE vs. DE	Indirect	Moderate	Imprecision^4^
AE vs. ARE	Indirect	Moderate	Imprecision^4^
AE vs. MCE	Mixed	Low	Inconsistency^1^; Imprecision^4^
RE vs. MBE	Indirect	Low	Indirectness^3^; Imprecision^4^
RE vs. DE	Indirect	Low	Indirectness^3^; Imprecision^4^
RE vs. ARE	Mixed	Very low	Inconsistency^1^^,^^2^; Imprecision^4^
RE vs. MCE	Mixed	Low	Inconsistency^1^; Imprecision^4^
MBE vs. DE	Indirect	Low	Indirectness^3^; Imprecision^4^
MBE vs. ARE	Indirect	Low	Indirectness^3^; Imprecision^4^
MBE vs. MCE	Mixed	Moderate	Imprecision^4^
DE vs. ARE	Indirect	Low	Indirectness^3^; Imprecision^4^
DE vs. MCE	Mixed	Moderate	Imprecision^4^
ARE vs. MCE	Mixed	Low	Inconsistency^1^; Imprecision^4^
Ranking of treatments	Network estimate	High	None

1 Downgraded due to significant local inconsistency identified in loop-specific inconsistency analysis.

2 Downgraded due to statistically significant disagreement between direct and indirect evidence identified by node-splitting analysis.

3 Downgraded due to indirect evidence only and concerns regarding the transitivity assumption.

4 Downgraded due to wide confidence intervals crossing the line of no effect.

### 3.4 Effects of different exercise modalities on global cognitive function in patients with MCI

A total of 55 studies involving 4,603 participants were included in the pooled analysis of global cognitive function. Cognitive outcomes were assessed using standardized instruments, including MMSE, MoCA, ADAS-Cog, ADAS-Cog–Executive Function, and MDRS. A total of six exercise modalities were examined for their effects on cognitive function in individuals with MCI. Specifically, 15 studies investigated AE, 7 evaluated RE, 14 assessed MBE, 10 examined DE, 7 evaluated combined ARE, and 15 assessed MCE. Control conditions were further categorized as AC or PC.

No statistically significant inconsistency was identified using either the loop-specific approach or the node-splitting method. The global inconsistency test further supported the assumption of consistency across the network (*χ*^2^(16) = 13.38, *p* = 0.6447). Therefore, the consistency model was adopted for the primary network meta-analysis. Detailed inconsistency assessments are presented in Appendix 3.

**Figure 3:**
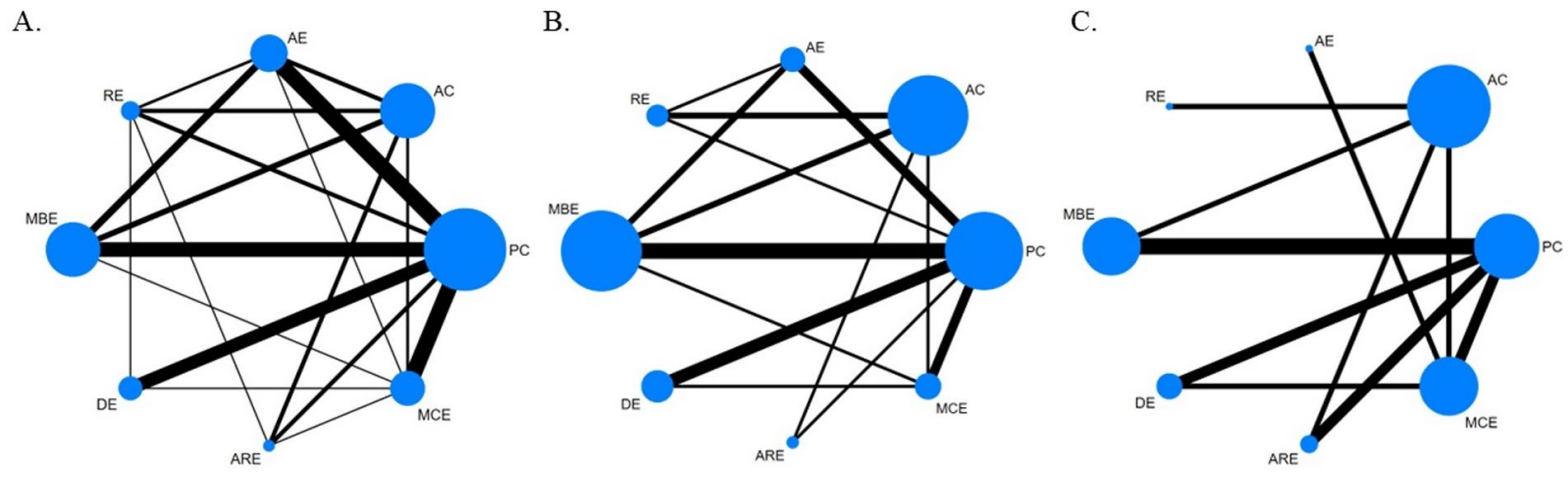
Network geometry of exercise interventions for cognitive outcomes in patients with mild cognitive impairment (MCI).

**Figure 4:**
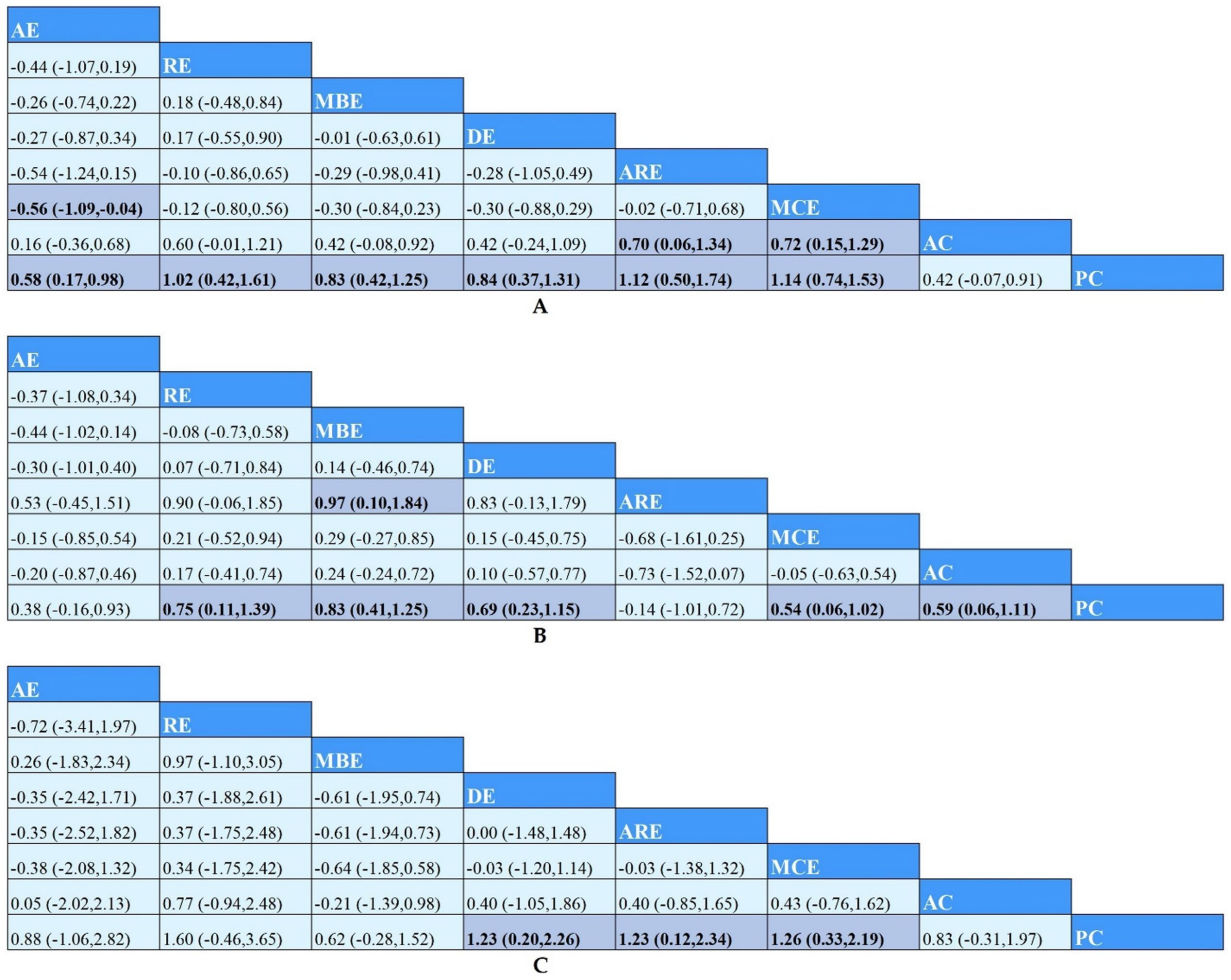
League tables of network meta-analysis comparing different exercise modalities for cognitive outcomes in patients with mild cognitive impairment (MCI). **(A)** Global cognitive function, **(B)** Cognition-related memory function, **(C)** Cognition-related executive function. Exercise modalities include aerobic exercise (AE), resistance exercise (RE), mind–body exercise (MBE), dance exercise (DE), combined aerobic and resistance exercise (ARE), multicomponent exercise (MCE), active control (AC), and passive control (PC). Values represent standardized mean differences (SMDs) with 95% confidence intervals (CIs) derived from the network meta-analysis based on change scores. Comparisons are read from the row-defining intervention versus the column-defining intervention. Dark green cells indicate statistically significant differences between interventions (95% CI not crossing zero), whereas light green cells indicate non-significant differences.

The network geometry of direct comparisons for global cognitive function is presented in Figure 3A. Network meta-analysis demonstrated that all exercise modalities were significantly more effective than PC in improving global cognitive function. In addition, ARE and MCE showed significantly greater effects than AC (Figure 4A).

**Figure 5:**
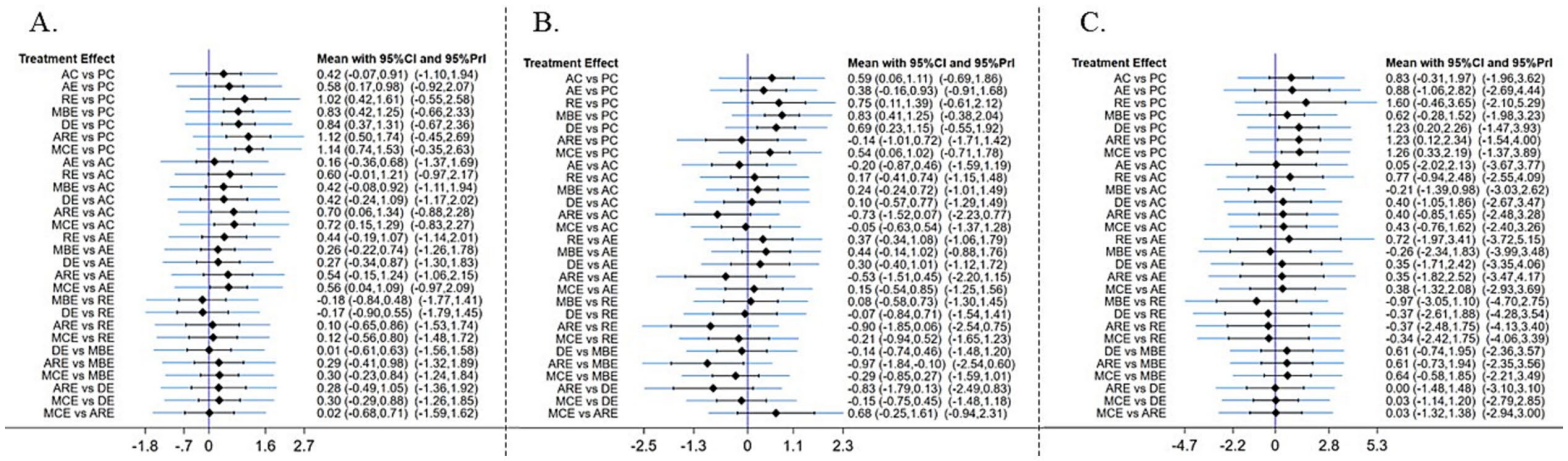
Comparative effects of different exercise modalities on cognitive outcomes in patients with mild cognitive impairment (MCI). Forest plots presenting the comparative effects of different exercise modalities and control conditions on cognitive outcomes based on network meta-analysis. Effect sizes are expressed as standardized mean differences (SMDs) with 95% confidence intervals (CIs). **(A)** Global cognitive function, **(B)** Cognition-related memory function, **(C)** Cognition-related executive function. Exercise modalities include aerobic exercise (AE), resistance exercise (RE), mind–body exercise (MBE), dance exercise (DE), combined aerobic and resistance exercise (ARE), and multicomponent exercise (MCE). Control conditions include passive control (PC) and active control (AC). Positive SMD values indicate favorable effects on cognitive outcomes. Comparisons with 95% CIs not crossing zero are considered statistically significant.

**Figure 6:**
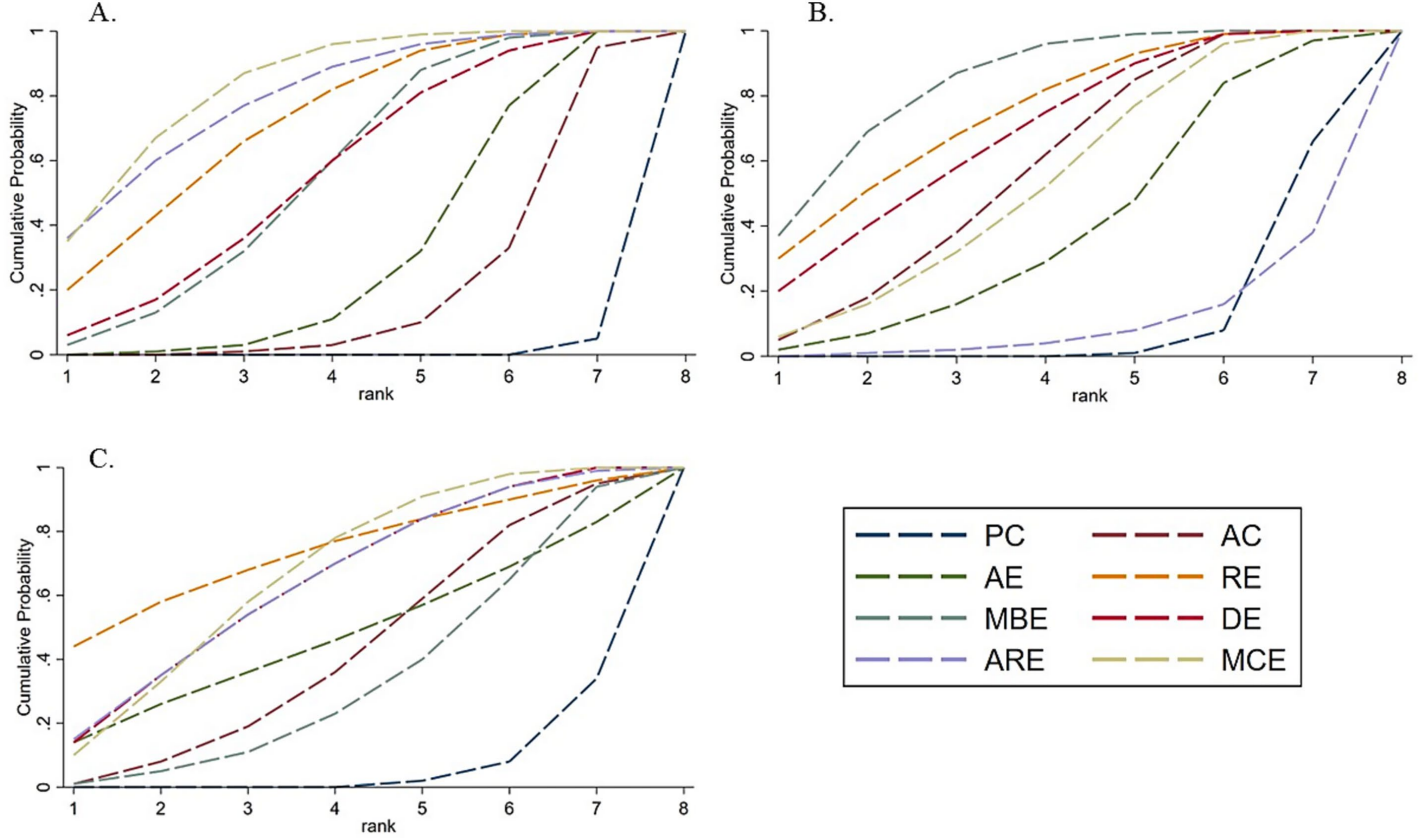
Cumulative ranking probabilities and SUCRA values of exercise modalities for cognitive outcomes in patients with mild cognitive impairment (MCI). **(A)** Global cognitive function, **(B)** Cognition-related memory function, **(C)** Cognition-related executive function. Exercise modalities include aerobic exercise (AE), resistance exercise (RE), mind–body exercise (MBE), dance exercise (DE), combined aerobic and resistance exercise (ARE), and multicomponent exercise (MCE). Control conditions include passive control (PC) and active control (AC).

The comparative effects among exercise modalities for global cognitive function are presented in Figure 5A. Intervention ranking for global cognitive function based on cumulative ranking probabilities and SUCRA values is shown in Figure 6A. Among all interventions, MCE demonstrated the highest probability of being the optimal intervention for improving global cognitive function, with a SUCRA value of 83.3%.

The robustness of the primary findings was further evaluated through sensitivity analyses using different assumed baseline–post-intervention correlation coefficients (*r* = 0.3, 0.7, and 0.9) for the imputation of change-score standard deviations. The overall network estimates and SUCRA-based treatment rankings remained largely unchanged across different assumptions, indicating that the primary findings were stable and not materially influenced by the choice of correlation coefficient. Detailed sensitivity analysis results are provided in Appendix 4.

Network plots illustrate the geometry of evidence for (A) global cognitive function, (B) cognition-related memory function, and (C) cognition-related executive function. Each node represents an exercise intervention or control condition, including passive control (PC), active control (AC), aerobic exercise (AE), resistance exercise (RE), mind–body exercise (MBE), dance exercise (DE), combined aerobic and resistance exercise (ARE), and multicomponent exercise (MCE). Node size is proportional to the total number of participants allocated to each intervention across included studies. Lines between nodes indicate direct head-to-head comparisons, and line thickness reflects the number of studies contributing to each comparison.

### 3.5 Exercise prescription characteristics of multicomponent exercise associated with global cognitive function

Given the superior ranking of MCE in the network meta-analysis, further pairwise meta-analyses were conducted to explore which exercise prescription characteristics were associated with greater improvements in global cognitive function. Random-effects models were used to compare MCE with control conditions, and prespecified subgroup analyses were performed according to exercise intensity, intervention period, training frequency, and session duration (Table 3).

**Table 3:** Subgroup analyses of exercise prescription characteristics of multicomponent exercise for global cognitive function.

Subgroup	Study number	Sample size	Test of overall effect			
			SMD (95%CI)	Z	*P*	Test for subgroup differences (*P*)
Intensity						
Low	3	364	0.153 (−0.639, 0.944)	0.378	0.705	0.040
Moderate	10	676	1.158 (0.616, 1.699)	4.190	<0.001	
Length of intervention						
Short (≤12 weeks)	5	302	0.796 (0.083, 1.508)	2.189	0.029	0.085
Moderate (13–24 weeks)	6	410	1.285 (0.384, 2.186)	2.796	0.005	
Long (≥25 weeks)	2	328	0.174 (−0.356, 0.705)	0.644	0.519	
Frequency						
Low (1–2 times/week)	3	160	0.413 (0.037, 0.788)	2.154	0.031	<0.001
Moderate (3–4 times/week)	7	676	0.454 (0.045, 0.863)	2.175	0.03	
High (≥5 times/week)	3	204	2.344 (1.5, 3.188)	5.443	<0.001	
Duration per session						
Short (≤45 min)	3	276	1.625 (0.326, 2.924)	2.452	0.014	0.220
Moderate (46–60 min)	7	566	0.497 (0.032, 0.961)	2.094	0.036	
Long (≥61 min)	3	204	1.122(−0.254, 2.499)	1.599	0.11	

*p*-values within each subgroup represent tests of the pooled effect. “Test for subgroup differences (P)” represents the between-subgroup comparison.

Overall, MCE was associated with significantly greater improvements in global cognitive function compared with control conditions. Subgroup analysis by exercise intensity showed a statistically significant difference between low- and moderate-intensity MCE (*p* = 0.04), with moderate-intensity MCE producing a larger effect estimate than low-intensity MCE. The pooled effect was significant in the moderate-intensity subgroup, whereas the low-intensity subgroup did not reach statistical significance.

When stratified by intervention period, the difference among short-term (≤12 weeks), mid-term (13–24 weeks), and long-term (≥25 weeks) interventions did not reach statistical significance (*p* = 0.085). Mid-term interventions lasting 13–24 weeks showed the largest effect estimate. However, because the between-subgroup difference did not reach statistical significance (*p* = 0.085), this finding should be interpreted as exploratory and hypothesis-generating rather than evidence of a definitive intervention-period effect.

Subgroup analysis by training frequency demonstrated significant differences among low-frequency (1–2 sessions/week), moderate-frequency (3–4 sessions/week), and high-frequency (≥5 sessions/week) MCE programs (*p* < 0.001). High-frequency interventions, defined as ≥5 sessions per week, showed the most pronounced effect on global cognitive function.

In contrast, subgroup analysis by session duration showed no statistically significant difference among short-duration (≤45 min), moderate-duration (46–60 min), and long-duration (≥61 min) sessions (*p* = 0.22). Although the ≤45-min subgroup showed the largest effect estimate, the between-subgroup difference was not statistically significant. Therefore, this finding should be interpreted as exploratory and hypothesis-generating rather than evidence of a definitive session-duration effect.

Overall, these findings suggest that moderate-intensity MCE performed at least five times per week was associated with greater improvements in global cognitive function among patients with MCI. Although interventions lasting 13–24 weeks and sessions of ≤45 min showed the largest effect estimates, respectively, neither intervention period nor session duration was identified as a statistically significant moderator. Therefore, these findings should be interpreted as exploratory and hypothesis-generating rather than definitive exercise prescription recommendations.

### 3.6 Effects of different exercise modalities on cognition-related memory function in patients with MCI

A total of 21 studies involving 1,785 participants were included in the pooled analysis of cognition-related memory function. In this study, Cognition-related memory outcomes included episodic and delayed memory measures, such as AVLT delayed recall, logical and story memory tests (e.g., WMS Logical Memory II, LMT), behavioral and visual memory tests (e.g., RBMT, BVRT, RBANS visual delayed recall), fixed-interval delayed recall tasks (10 or 30 min), composite memory indices (e.g., WMS-MQ), and digit span backward as a measure of working memory storage.

A total of six exercise modalities were examined for their effects on memory-related outcomes in individuals with MCI. Specifically, 3 studies investigated AE, 3 assessed RE, 7 examined MBE, 5 evaluated DE, 2 investigated ARE, and 5 assessed MCE. Control conditions were categorized as AC or PC.

No statistically significant inconsistency was detected using either the loop-specific approach or the node-splitting method. The global inconsistency test further supported the assumption of consistency across the network (*χ*^2^(8) = 14.68, *p* = 0.0657). Accordingly, the consistency model was considered appropriate and was applied in the primary network meta-analysis. Detailed inconsistency assessment results are provided in Appendix 3.

The network geometry of direct comparisons for memory-related outcomes is presented in Figure 3B. Network meta-analysis demonstrated that RE, MBE, DE, and MCE were significantly more effective than PC in improving memory-related outcomes (Figure 4B).

The comparative effects among exercise modalities are presented in Figure 5B. Intervention ranking based on cumulative ranking probabilities and SUCRA values is shown in Figure 6B. Among all interventions, MBE demonstrated the highest probability of being the optimal intervention for improving memory-related outcomes, with a SUCRA value of 83.9%. However, due to the limited number of eligible studies and the relatively lower certainty of evidence for several indirect comparisons, additional pairwise meta-analyses and subgroup analyses focusing specifically on MBE were not conducted.

### 3.7 Effects of different exercise modalities on cognition-related executive function in patients with MCI

Executive-function-related outcomes included performance-based measures of attention and processing speed (e.g., TMT-A and DSST), executive control and cognitive flexibility (e.g., TMT-B, TMT B–A difference, and task-switching paradigms), inhibitory control (e.g., Stroop interference indices and d2 concentration index), working memory (e.g., digit span backward and n-back tasks), and verbal executive retrieval (e.g., letter and category verbal fluency tests). When multiple executive-related measures were reported within a study, outcomes were directionally aligned and synthesized using z-score aggregation.

Given the heterogeneity of executive test batteries across trials, executive outcomes were synthesized at the study level. Within each study, all eligible executive-related measures were directionally aligned and standardized, and then combined into a single composite score using z-score aggregation, such that each study contributed one effect size for cognition-related executive functioning. These study-level composites were subsequently pooled using SMDs within a random-effects network meta-analysis framework. The resulting estimates therefore represent average effects on executive-related cognitive performance rather than effects on a single latent executive function construct.

A total of 15 studies involving 1,481 participants were included in the pooled analysis of executive-function-related outcomes. A total of six exercise modalities were examined. Specifically, 1 study investigated AE, 1 evaluated RE, 4 assessed MBE, 3 examined DE, 3 evaluated combined ARE, and 5 assessed MCE. Control conditions were categorized as AC or PC.

No statistically significant inconsistency was identified using either the loop-specific approach or the node-splitting method. The global inconsistency test further supported the assumption of consistency across the network (*χ*^2^(3) = 0.63, *p* = 0.8888). Therefore, the consistency model was adopted for the primary network meta-analysis. Detailed inconsistency assessment results are presented in Appendix 3.

The network geometry of direct comparisons for executive-function-related outcomes is presented in Figure 3C. Network meta-analysis demonstrated that AE, RE, DE, ARE, and MCE were significantly more effective than PC in improving executive-function-related outcomes. Network meta-analysis demonstrated that AE, RE, DE, ARE, and MCE were significantly more effective than PC in improving executive-function-related outcomes, all exercise interventions were directly compared with control conditions within the executive-function network (Figure 4C).

The comparative effects among exercise modalities are presented in Figure 5C. Intervention ranking based on cumulative ranking probabilities and SUCRA values is shown in Figure 6C. Among all interventions, RE demonstrated the highest probability of being the optimal intervention for improving executive-function-related outcomes, with a SUCRA value of 73.7%. However, these findings should be interpreted cautiously because several exercise modalities were represented by only a limited number of studies, and the certainty of evidence for some indirect comparisons was relatively low. Accordingly, additional pairwise meta-analyses and subgroup analyses focusing specifically on RE were not conducted (see Figure 6).

## 4 Discussion

### 4.1 Principal findings

In this network meta-analysis of 55 randomized controlled trials involving 4,603 participants with MCI, exercise interventions were generally associated with improvements across cognitive domains when compared with passive control. Among exercise modalities, MCE demonstrated the most favorable ranking for global cognitive function, whereas MBE ranked highest for memory-related outcomes and RE showed the highest ranking probability for executive-function-related outcomes. These findings suggest that different exercise modalities may preferentially benefit distinct cognitive domains rather than exerting uniform effects across all aspects of cognition.

The network showed no statistically significant inconsistency across global cognition, cognition-related memory, or cognition-related executive function outcomes, supporting the appropriateness of the consistency model for the primary analyses. This strengthens confidence in the internal coherence of the relative effect estimates derived from both direct and indirect evidence within the network.

### 4.2 Interpretation by cognitive domain: why different modalities may favor different outcomes

#### 4.2.1 Global cognitive function: why MCE may rank highest

MCE may have ranked highest for global cognitive function because it typically integrates aerobic, resistance, balance/coordination, and sometimes cognitive or dual-task elements. Such multimodal stimulation plausibly engages broader neurocognitive networks than single-modality training, which may translate into more consistent gains on global screening measures (e.g., MMSE, MoCA, ADAS-Cog) that capture multiple cognitive subdomains.

The cognitive benefits of multicomponent exercise in individuals with MCI are likely mediated by the complementary effects of its distinct training components. The aerobic component may enhance cerebral perfusion and metabolic efficiency, thereby supporting neuronal energy homeostasis and creating a favorable neurobiological environment for cognitive functioning. Although increases in brain-derived neurotrophic factor (BDNF) are frequently proposed as a potential mechanism, recent evidence suggests that cognitive improvements following multicomponent interventions may occur independently of detectable changes in circulating BDNF levels, indicating the involvement of alternative vascular and metabolic pathways (Lam et al., 2015). In addition, the resistance and strength-training components may contribute to cognitive benefits through improvements in neuromuscular coordination and anabolic signaling, such as insulin-like growth factor-1–related pathways, which are closely linked to the maintenance of frontoparietal executive networks (Lam et al., 2015; Nascimento et al., 2014). Importantly, balance- and coordination-based elements impose substantial cognitive and attentional demands, thereby increasing prefrontal cortical engagement. This heightened cognitive load may directly facilitate executive control processes, including task switching, inhibitory control, and working memory. The integration of aerobic, resistance, and cognitively demanding motor tasks may therefore produce synergistic effects on both memory- and executive-related cognitive outcomes in individuals with MCI (Bademli et al., 2019; De Sa et al., 2024).

#### 4.2.2 Cognition-related memory: why MBE may show comparative advantages

Accumulating evidence suggests that MBE may exert particularly pronounced benefits on cognitive function, especially cognition-related memory, in individuals with MCI through multiple complementary mechanisms. Neuroimaging studies indicate that mind–body interventions such as Tai Chi and Baduanjin can directly modulate memory-related neural circuits, including the hippocampus, anterior cingulate cortex, and angular gyrus, with observed increases in gray matter volume, regional spontaneous neural activity, and functional connectivity within default mode and memory-related networks—changes that are closely linked to episodic and delayed memory performance (Lam et al., 2011; Tao et al., 2019). In parallel, MBE is characterized by sustained attentional engagement, complex movement sequencing, and continuous sensorimotor integration, which place higher cognitive demands on prefrontal control systems than conventional aerobic exercise alone; enhanced prefrontal activation and cerebral oxygenation during Tai Chi practice have been associated with better memory encoding and retrieval processes (Lin et al., 2025). At the molecular level, randomized controlled trials have demonstrated that Tai Chi-based interventions can upregulate circulating BDNF, a key mediator of synaptic plasticity and hippocampal-dependent learning and memory, providing a plausible biological pathway linking MBE to memory-specific cognitive gains (Lin et al., 2025).

Moreover, the intrinsic “cognitive–motor coupling” of MBE—requiring sustained attention, action monitoring, and internal representation of movement sequences—may function as an implicit form of cognitive training, thereby preferentially enhancing memory consolidation and recall compared with more monotonous exercise modalities (Lam et al., 2011; Liu et al., 2021). Collectively, these converging neurobiological, neuroimaging, and behavioral mechanisms provide a coherent explanation for why MBE demonstrated the highest probability of benefit for cognition-related memory outcomes in the present network meta-analysis.

#### 4.2.3 Cognition-related executive function: why RE may rank highest

RE demonstrated the highest probability of benefit for cognition-related executive function, which may be attributable to the unique neurophysiological and cognitive demands associated with resistance-based training. Executive function relies heavily on prefrontal and frontoparietal neural networks responsible for inhibitory control, attentional regulation, cognitive flexibility, and working memory. Compared with more automated aerobic activities, resistance exercise often requires continuous motor planning, force regulation, movement monitoring, and coordination of complex multi-joint actions, thereby imposing greater demands on executive control systems (Jerez-Salas et al., 2025; Chan et al., 2022).

Emerging neuroimaging and behavioral evidence suggests that resistance exercise is associated with increased activation of prefrontal cortical regions and enhanced functional connectivity within executive-control networks (Hortobágyi et al., 2022). In addition, resistance training may preferentially influence neurobiological pathways implicated in executive functioning, including insulin-like growth factor-1 (IGF-1), catecholaminergic signaling, and lactate-mediated neurometabolic adaptations, all of which are closely linked to prefrontal cortical efficiency and synaptic plasticity (Cassilhas et al., 2007; Hwang D. et al., 2023). These mechanisms may be particularly relevant in individuals with MCI, in whom executive dysfunction is commonly associated with frontal lobe vulnerability and impaired cognitive control processes.

Furthermore, resistance exercise typically involves externally guided task execution, repetition monitoring, posture stabilization, and rapid adjustment to changing mechanical demands. Such cognitively demanding motor regulation may function as an implicit form of executive training, thereby enhancing inhibitory control and task-switching abilities beyond the effects observed with less cognitively engaging exercise modalities (Jerez-Salas et al., 2025; Chan et al., 2022; Chen et al., 2020). Collectively, these neurocognitive and neuromuscular mechanisms may explain why RE demonstrated the highest ranking probability for cognition-related executive outcomes in the present network meta-analysis.

### 4.3 Exercise prescription implications for MCE (global cognition function)

An important contribution of this review is the exploration of exercise prescription characteristics for MCE in relation to global cognitive function. Pairwise meta-analysis and prespecified subgroup analyses suggested that MCE was associated with significant improvements compared with controls, and that intervention period and training frequency may meaningfully influence observed effects. Specifically, interventions lasting 13–24 weeks and delivered at ≥5 sessions per week were associated with the most pronounced improvements, whereas session duration and intensity showed less consistent between-subgroup differences, despite more favorable point estimates for moderate intensity and shorter sessions (≤45 min).

These findings should be interpreted cautiously. Subgroup analyses in meta-analysis are observational by design and may be influenced by confounding across intervention characteristics (e.g., longer programs may also differ in supervision, adherence, or baseline cognitive severity). Therefore, the suggested prescription profile should be viewed as hypothesis-generating and supportive of program design, rather than definitive evidence of a causal dose–response relationship.

### 4.4 Comparison with previous evidence

Previous systematic reviews and meta-analyses have consistently reported that exercise interventions exert beneficial effects on cognitive function in individuals with MCI, supporting exercise as a key non-pharmacological strategy for delaying cognitive decline (Liu et al., 2024; Lin et al., 2023; Wang et al., 2019). Network meta-analyses focusing on global cognition have further suggested that multicomponent exercise and combined aerobic–resistance training may rank among the most effective modalities for overall cognitive improvement (Yu et al., 2024; Wang et al., 2019).

However, much of the existing evidence has primarily concentrated on global cognitive outcomes or on single exercise modalities, such as walking, Tai Chi, or resistance training, without systematically differentiating effects across specific cognitive domains (Lin et al., 2023; Yu et al., 2021; Shao et al., 2024). As a result, whether different exercise modalities preferentially benefit distinct cognitive functions—such as memory versus executive control—has remained insufficiently addressed in prior syntheses (Yu et al., 2024; Liu et al., 2024).

Several meta-analyses have reported favorable effects of MBE, particularly Tai Chi and Baduanjin, on memory-related outcomes in MCI populations, highlighting improvements in episodic memory and delayed recall performance (Yu et al., 2021; Shao et al., 2024). These findings are broadly consistent with the present results, which indicate that MBE shows comparatively greater benefits for cognition-related memory function. However, previous studies have typically evaluated MBE in isolation, rather than within a comprehensive comparative framework that includes multiple exercise modalities (Yu et al., 2021; Shao et al., 2024).

In contrast to earlier work, the present network meta-analysis simultaneously compared multiple exercise modalities across three distinct cognitive domains—global cognition, cognition-related memory, and cognition-related executive function. This domain-specific approach extends previous evidence by demonstrating that multicomponent exercise is most strongly associated with improvements in global cognitive function and cognition-related executive function, whereas MBE appears to preferentially target memory-related outcomes. Such differentiation provides a more nuanced understanding than prior analyses that relied on a single pooled cognitive endpoint (Yu et al., 2024; Wang et al., 2019; Gómez-Soria et al., 2022).

Moreover, while some recent network meta-analyses have explored optimal exercise types or doses for cognitive improvement in MCI, few have conducted additional pairwise and subgroup analyses to translate ranking results into practical exercise prescriptions (Yu et al., 2024; Gómez-Soria et al., 2022). By further examining exercise intensity, frequency, session duration, and intervention period for multicomponent exercise, the present study adds actionable evidence to the existing literature and complements prior findings that emphasized efficacy ranking without detailed prescription guidance (Yu et al., 2024; Gómez-Soria et al., 2022).

Overall, the present findings are largely concordant with previous evidence supporting the cognitive benefits of exercise in MCI, while advancing the field by clarifying domain-specific effects and providing empirically grounded guidance for multicomponent exercise prescription. This comparative and domain-oriented perspective represents a meaningful extension of earlier meta-analytic work and contributes to a more targeted, evidence-informed framework for exercise-based cognitive intervention in MCI (Yu et al., 2024; Liu et al., 2024; Wang et al., 2019).

### 4.5 Strengths

This study has several methodological and analytical strengths. First, a network meta-analysis framework was applied to enable simultaneous comparison of multiple exercise modalities and to integrate direct and indirect evidence. Second, cognitive outcomes were analyzed by domain (global cognition, cognition-related memory, and cognition-related executive function), allowing a more nuanced evaluation of modality-specific effects than would be possible using a single pooled cognitive endpoint.

Importantly, different outcome synthesis strategies were applied for memory and executive domains to reflect their distinct psychometric structures while maintaining one independent effect size per study for each domain. For cognition-related memory, when multiple memory measures were reported within a study, one prespecified and most representative outcome—typically an episodic or delayed recall measure—was selected *a priori* to avoid double counting and preserve statistical independence, in line with recommendations for meta-analysis of correlated outcomes. In contrast, cognition-related executive function was treated as a multidimensional construct. Given the substantial heterogeneity of executive test batteries across trials and the frequent reporting of multiple executive-related outcomes within individual studies, all eligible executive measures were directionally aligned and standardized, and then aggregated into a single study-level composite score using z-score transformation (Song et al., 2013). This approach enabled standardized synthesis of fragmented executive outcomes and facilitated domain-level comparison across studies, albeit at the cost of collapsing potentially distinct executive subcomponents.

Third, inconsistency assessments at both global and local levels did not indicate meaningful incoherence, supporting the robustness of the network structure. Finally, the additional exploration of exercise prescription characteristics for multicomponent exercise provides clinically relevant signals for exercise program design in individuals with MCI.

### 4.6 Limitations

Several limitations should be acknowledged. First, the number of studies available for cognition-related memory and executive outcomes was modest, and some exercise modalities were represented by relatively few trials, which may limit precision and increase susceptibility to small-study effects. Second, risk of bias was not uniformly low across all domains, and uncertainty—particularly in allocation concealment—may have influenced some estimates. Third, intervention protocols varied substantially across trials in terms of intensity definitions, supervision, adherence, and co-interventions, which may contribute to residual heterogeneity despite the use of random-effects models. Finally, subgroup analyses of multicomponent exercise prescription characteristics were based on between-study comparisons and should therefore be interpreted as exploratory rather than confirmatory.

### 4.7 Implications for practice and research

From a practical standpoint, these findings support exercise as a beneficial non-pharmacological strategy for cognitive health in MCI, with modality selection potentially guided by the targeted cognitive domain. Where the goal is global cognitive improvement or cognition-related executive function, MCE may be prioritized; where episodic memory is the primary target, MBE may be considered. Program selection should also incorporate feasibility, comorbidities, safety, and patient preference to promote adherence.

Future research should prioritize adequately powered, well-reported randomized controlled trials with standardized cognitive outcome selection and transparent reporting of exercise dose parameters (frequency, intensity, time, and duration). Head-to-head trials comparing MCE and MBE and trials that explicitly manipulate prescription variables would be particularly valuable to test the hypothesis that different modalities preferentially affect distinct cognitive domains and to establish more definitive prescription recommendations.

## 5 Conclusion

This network meta-analysis demonstrates that different exercise modalities may exert domain-specific cognitive benefits in individuals with MCI. Multicomponent exercise was associated with the most favorable effects on global cognitive function, whereas MBE showed comparatively greater benefits for memory-related outcomes and RE demonstrated the highest ranking probability for executive-function-related outcomes. These findings suggest that exercise interventions may differentially target distinct cognitive domains rather than producing uniform cognitive effects.

Due to methodological constraints and the limited number of available studies, additional pairwise meta-analyses and subgroup analyses were conducted only for global cognitive function, for which multicomponent exercise ranked highest.

Based on these analyses, moderate-intensity multicomponent exercise performed at least five times per week was associated with greater improvements in global cognitive function among individuals with MCI. Interventions lasting 13–24 weeks and sessions of ≤45 min showed larger effect estimates; however, neither intervention period nor session duration was identified as a statistically significant moderator. Therefore, these findings should be interpreted as exploratory and hypothesis-generating rather than definitive exercise prescription recommendations.

Nevertheless, all examined exercise modalities demonstrated varying degrees of beneficial effects on cognition, memory, and executive function. Accordingly, exercise selection in clinical and community settings should also consider individual preferences, physical capacity, safety, and feasibility to enhance long-term adherence and implementation.

Future studies should further clarify modality-specific cognitive effects and establish evidence-based exercise prescription recommendations for individuals with MCI.

## Data Availability

The original contributions presented in the study are included in the article/supplementary material, further inquiries can be directed to the corresponding authors.
