# Supplementary material for: Exercise modalities in mild cognitive impairment: a systematic review and network meta-analysis of comparative effectiveness
Source: Front Aging Neurosci. 2026 Jul 16;18:1888709. doi: 10.3389/fnagi.2026.1888709 (PMC13422556; doi:10.3389/fnagi.2026.1888709)
Supplement: Supplementary file 1 [file Table_1.pdf]

Appendix 1A

Search strategy : (Mild cognitive impairment OR MCI OR "mild cognitive impairment, amnestic type" OR "mild cognitive impairment, non-amnestic type") AND (physical activity OR exercise OR "bodily activity" OR "aerobic exercise" OR MICT OR HIIT OR HICT OR "resistance training" OR "strength training" OR "flexibility training" OR "balance training" OR "home-based exercise" OR "rehabilitation exercise" OR "aquatic rehabilitation" OR "Tai Chi" OR "dance" OR "martial arts") AND ("cognitive function" OR cognition OR "cognitive ability" OR "activities of daily living" OR ADL OR "instrumental activities of daily living" OR IADL) AND (randomized controlled trial OR RCT OR "randomised controlled trial")

. Appendix 1B Detailed intervention characteristics and cognitive outcome measures contributing to the network meta-analyses

Study	Control category	Exercise category	Control description	Intervention description	Global cognition outcome included in NMA	Memory outcome included in NMA	Executive -function outcomes included in NMA
Bademli 2018[27]	PC	MCE	No exercise intervention: Maintain daily activities without any exercise or cognitive training.<br>Intervention period: Observation for 20 weeks concurrently. After the intervention, the control group was provided with the same physical activity program.	1. Warm-up (10 minutes, muscle and joint activation);<br>2. Rhythmic exercises (20 minutes, 22 simple movements, stretching + endurance training);<br>3. Cool-down (10 minutes, to lower heart rate and blood pressure);<br>4. Free walking (40 minutes, recorded with a pedometer);<br>Implementation method: Researchers will guide groups (5 groups of 6 people each), primarily group training.	SMMSE		
Bisbe 2020[28]	-	DE MCE	Active Exercise Control (No Cognitive Confounding):<br>The control group received "Multimodal Physical Therapy" (exercise intervention alone), providing a contrast between the "Choreographed Dance" group and other exercise types;<br>There was no intervention-free control group; the core focus was on a direct comparison of the two exercise methods.	Two separate exercise experimental groups (neither with cognitive training):<br>1. Dance group: Choreographic aerobic dance (salsa, rock, etc.), with the core focus on learning dance sequence aerobic training.<br>2. Physical therapy group: Multimodal physical therapy, including strength, balance, coordination, and gait training (using resistance bands, balance mats, etc.); Neither group received additional cognitive training; both were purely exercise interventions, differing only in the type of exercise.	MMSE；	RBANS	VDR； TMT-A； TMT-B； LVFT； CVFT
Chang 2021[29]	PC	DE	No specific sports activities will be arranged, and voluntary daily activities will not be restricted; a regular lifestyle will be maintained.	Chinese square dancing (aerobic group exercise): includes movements such as clapping, hand-clapping, chest expansion, arm stretching, and leg kicking, all in sync with music rhythm. The	MoCA		

Study	Control category	Exercise category	Control description	Intervention description	Global cognition outcome included in NMA	Memory outcome included in NMA	Executive -function outcomes included in NMA
				**movements are simple and easy to learn.**			
**DeSá 2023[30]**	AC	ARE MCE	**No-exercise control (exercise task only):<br>1. Intervention content: Gentile-based exercise task (balance and coordination training, no physical load, not involving aerobic/strength exercise);<br>2. No physical exercise component, intervention duration and frequency matched to the PE group.**	**Exercise-only experimental group (PE group, without cognitive confounding):<br>1. Exercise type: Multimodal physical exercise, primarily aerobic training combined with strength training (covering major muscle groups);<br>2. No cognitive training or exercise task components, pure exercise intervention.**	MMSE		
**Doi 2017[31]**	PC	DE	**Attend 3 health education training sessions (90 minutes each) within 40 weeks, covering topics such as aging, fall prevention, healthy eating, oral care, and frailty management; mail 3 relevant topical handbooks (without cognitive/physical activity information); conduct telephone follow-ups to improve adherence, without restricting self-consciousness/physical activity.**	**Intervention Group 1: Dance (social dance, including salsa, rumba, waltz, cha-cha, blues, jitterbug, tango); Intervention Group 2: Playing percussion instruments (such as conga drums)**	MMSE	Story Memory	TMT-A；<br>TMT-B
**Fiatarone Singh 2014[32]**	AC	RE	**Double-control program:<br>1. Pseudo-cognitive training: Watching a National Geographic short film + answering questions (no feedback, minimizing cognitive stimulation);<br>2. Pseudo-exercise training: Stretching, seated aerobics (low intensity, does not increase heart rate, muscle strength, or balance);<br>3. Session duration: 60 minutes;<br>4. Frequency: Initially 3 times/week, later adjusted to 2 times/week;<br>5. Intervention period: 6 months**	**High-Intensity Progressive Resistance Training (PRT):<br>1. Core Equipment: Pneumatic resistance machine;<br>2. Training Movements: 5-6 movements including chest press, leg press, seated row, standing hip abduction, and knee extension (covering major muscle groups);<br>3. Training Mode: 3 sets x 8 repetitions/movement**	ADAS-Cog	BVRT	WAIS-III Similarities；<br>WAIS-III Matrices；<br>LVFT；CVFT
**Fonte 2019[33]**	PC	ARE	**Standard drug therapy (cholinesterase inhibitors, etc.), with no non-drug interventions (no**	**Intervention Group 2 (PT) received only exercise intervention (compound exercise: endurance + resistance training):**	MMSE	RBMT	TMT-A；<br>TMT-B；FAB

Study	Control category	Exercise category	Control description	Intervention description	Global cognition outcome included in NMA	Memory outcome included in NMA	Executive -function outcomes included in NMA
			exercise, no cognitive training).	1. Endurance training: treadmill walking, stationary bike, upper limb crank exercises (randomized sequence, 15 minutes each);<br>2. Resistance training: isotonic machine training such as chest press, seated lat pulldown, leg press (3 sets × 12 repetitions);<br>3. 15-minute warm-up (joint mobilization + treadmill walking) + stretching and relaxation (after training)			
Hwang 2023[34]	AC	MBE	Health Education (HE): Delivered once a week for 6 months (24 times in total);<br>Content: 15-minute stretching and flexibility exercises + 30-minute health lecture (promotion of nutrition, safety, exercise, and mental activity); No additional exercise or cognitive training components.	Tai Chi Group (TCE, Individual Exercise): 8-Form Yang Style Tai Chi, focusing on movement coordination, center of gravity control, and breathing coordination, with no additional cognitive training components; Implementation Method: Professional coach provides in-home guidance (over 10 years of Tai Chi experience), and participants are required to self-train at least 3 times per week after each intervention;	MDRS		
Jiang 2019[35]	PC	MCE	Routine Activities + Health Education Comparison:<br>1. Routine Activities: Activities at the community senior activity center (reading, chess, card games, singing and dancing, etc.);<br>2. Additional Interventions: Distribution of health manuals + health lectures;<br>3. No exercise intervention;<br>4. Intervention Period: 10 weeks	Motor intervention alone (no other intervention components):<br>1. Type of movement: Structured limb movement (combined upper and lower limb movements);<br>2. Upper limb movements: Pegboard insertion/removal, sandbag throwing, ball rotation/lifting/pressing;<br>3. Lower limb movements: Hopscotch (moving through a grid on the ground), alternating weight-bearing on toes and heels;<br>4. Pure motor intervention, without cognitive training, medication, or other components.	MoCA		
Khanthong 2021[36]	PC	MBE	No exercise intervention: Patients received only MCI and dementia prevention information from a neurologist at the beginning of the study (no additional exercise, cognitive training, or medication intervention); Intervention period: 12 weeks of concurrent observation.	Individual Exercise Experimental Group (RSD Group): Traditional Thai mind-body exercise Ruesi Dadton (15 movements), including slow movements, deep breathing, stretching, and balance training. It is a mindful physical exercise without additional cognitive training or medication.<br>Implementation Method: On-site guidance from professional Thai traditional medicine instructors	MoCA		TMT-A;<br>TMT-B

Study	Control category	Exercise category	Control description	Intervention description	Global cognition outcome included in NMA	Memory outcome included in NMA	Executive -function outcomes included in NMA
Khattak 2024[16]	PC	AE MCE	Isolated Exercise Control (Treadmill Walking):1. 20 minutes, 3 times a week, steady pace without incline; Intensity: Borg scale score 5-7 (moderate intensity);	(2 days of pre-training + 12 weeks of formal intervention), with local language instructional videos provided as supplementary support; primarily group training.<br>Individual Exercise Experimental Group (Multi-component Exercise): No mixed intervention, exercise intervention only;Exercise Types: Integrating aerobic training (multi-directional movement), strength training (upper and lower limbs + core muscles), balance training (static + dynamic), coordination and agility training (route practice), reaction time training (stomping, ball bouncing), and flexibility training (static stretching);<br>Implementation Method: Progressive in 3 stages (increasing intensity/number of sets), intensive training in the hospital's rehabilitation department, guided by professional physical therapists.	MoCA		
Kohanpour 2017[37]	PC	AE	Placebo controlled (no exercise/drug intervention):<br>1. Oral placebo capsules (without licorice extract);<br>2. No structured exercise training;<br>3. Maintenance of daily activities, no intervention in cognitive or motor function.	Isolated Exercise Experimental Group: Simple aerobic training (no confounding interventions); Exercise Format: Running (structured aerobic training), using a treadmill or running track; Implementation Method: Structured training guided by professionals, with gradually increasing duration; No drugs or cognitive training confounded.	MMSE		
Krootnark 2024[38]	PC	AE RE	Maintain daily activities and do not participate in any sports or cognitive training.	1. Aerobic Group: Low-intensity home aerobic exercise (indoor walking, stepping in place, multi-directional stepping, etc.); 2. Resistance Group: Low-intensity home resistance exercise (shoulder flexion and extension, wall push-ups, stepping up stairs, etc., using body weight or household items for support).	MoCA	DST-B	TMT-B;<br>SCWT III
Labott 2025[39]	PC	DE	Passive Control:<br>1. Does not accept dance or other structured interventions;<br>2. Maintains daily activities, prohibited from starting new cognitively intensive leisure activities	Individual Exercise Experimental Group (Dance Group): Structured dance intervention (no other interventions mixed in);<br>Exercise Format: Includes rhythmic movements, balance training, and limb coordination exercises, gradually increasing movement complexity,	MMSE		

Study	Control category	Exercise category	Control description	Intervention description	Global cognition outcome included in NMA	Memory outcome included in NMA	Executive-function outcomes included in NMA
			**or physical training;<br>3. May voluntarily participate in dance interventions as compensation after the intervention ends.**	**conducted on non-consecutive workdays;<br>Implementation Method: Community-based group training, guided by professionals, using standardized procedures.**			
**Lam 2010[40]**	AC	MBE	**Individual Exercise Control Group (Stretching and Conditioning Group):<br>1. Exercise Type: Muscle stretching and conditioning exercises designed by a physical therapist, with low cognitive requirements;<br>2. Implementation Method: Community group training, 8-12 week induction period (weekly instruction until mastery) + home practice, one review session per month, with video assistance provided;<br>3. Frequency/Duration: ≥3 times per week, ≥30 minutes per session, attendance recorded at the center, absence reminders provided.**	**Individual Exercise Experimental Group (Tai Chi Group): Moderate-to-low intensity physical and mental exercise, without cognitive training confounding effects;<br>Exercise Format: Simplified 24-form Tai Chi, taught by a Tai Chi master, 8-12 week induction period (weekly instruction until mastery) + home practice, monthly review sessions, video assistance provided;<br>Implementation Method: Community group training, central supervision, emphasizing movement coordination and concentration, suitable for MCI populations.**	CMMSE	Delayed Recall	CVFT；TMT-A(Chinese version)；TMT-B(Chinese version)；DST-B
**Lam 2014[41]**	AC	MBE	**Active Control (No Exercise Component):<br>1. Intervention Content: Stretching and relaxation exercises designed by a physical therapist;<br>2. No aerobic, balance, or cognitive stimulation components; focus solely on muscle stretching and relaxation;<br>3. Frequency and duration matched to the experimental group; confounding factors such as social contact were controlled.**	**24-Form Tai Chi**	MMSE	Delayed recall	
**Linda 2015[42]**	AC	MCE	**Active Control Group (Social Activity Group, S):<br>1. Low cognitive and physical demands social activities (tea parties, movies, shopping, etc.);**	**Individual Exercise Experimental Group (P): Multi-component low-to-moderate intensity exercise, without cognitive training mixing;<br>Exercise Format: 3 times per week, 1 hour each time, including 3 types of exercise rotation:**	CMMSE	Delayed Recall	CVFT；TMT-A(Chinese version)；TMT-B(Chinese

Study	Control category	Exercise category	Control description	Intervention description	Global cognition outcome included in NMA	Memory outcome included in NMA	Executive -function outcomes included in NMA
			2. Three times a week, one hour each time, conducted collectively at the community center; No exercise or high-intensity cognitive training components.	1. Stretching exercises;<br>2. Mind-body exercises (Tai Chi);<br>3. Aerobic exercise (static cycling, etc.);<br>Implementation Method: Group training at community center (at least once a week) + home practice (family supervision), staff record attendance and adherence.			version);<br>DST-B
Langoni 2018[43]	PC	ARE	No exercise intervention:<br>1. Maintain daily activities without introducing any new physical or cognitive training;<br>2. Regular telephone follow-ups to confirm no additional exercise participation;<br>Intervention period: 24 weeks, unsupervised exercise schedule	Individual Exercise Experimental Group: Aerobic + Strength Training Combination Exercise, without cognitive training confounding effects;<br>Exercise Format:<br>1. Aerobic Exercise: Walking (20-30 minutes, increasing duration with each cycle);<br>2. Strength Training: Using ankle weights, resistance bands, and dumbbells, covering movements such as elbow flexion/extension, shoulder abduction, and squats (progressively increasing sets/repetitions);<br>Implementation Method: Community group training, 5-8 people/group, led by a physical therapist + assisted by 2 assistants, each session includes warm-up/cool-down."	MMSE		
Lazarou 2017[44]	PC	DE	Maintain daily routine and do not participate in any intervention programs.	International Standard Ballroom Dancing (Aerobic + Social + Coordination Exercise): Group classes with professional instructors; requires partners; includes Tango, Waltz, Rumba, etc., and incorporates traditional Greek ballroom dances, with progressively more complex steps.	MoCA	RBMT 2	
Li 2021[45]	PC	MCE	1. Monthly community health guidance, 1 hour per session, covering general fitness-related knowledge;<br>2. Specific interventions such as unstructured exercise and cognitive training.	"Pure multi-component exercise (without cognitive tasks or other confounding factors):<br>1. Core exercise components: Aerobic training (shoulder movements, clapping, knee raises), strength training (half squats, bending and standing, elbow and knee bends), balance training (single-leg support, leg kicks), coordination training (three-step forward and backward movement, zigzag movement), agility training (fixed-pattern rapid foot clapping, ball-bouncing walking);	MoCA		

Study	Control category	Exercise category	Control description	Intervention description	Global cognition outcome included in NMA	Memory outcome included in NMA	Executive -function outcomes included in NMA
				2. Each session is 30 minutes, including 5 minutes of warm-up (stepping in place, static stretching) + 20 minutes of main exercises (5 minutes of each of the 5 exercises) + 5 minutes of cool-down (not explicitly stated, deduced from the intervention description);<br>3. Support: Provide an exercise manual and video to guide home practice."			
Li 2023[46]	AC	MBE	Low-intensity stretching exercises: full-body and localized (upper and lower limbs) stretching, joint mobilization, and diaphragmatic breathing;<br>Implementation method: delivered via virtual real-time video conferencing, 60 minutes/session, twice/week, for a total of 6 months;<br>No additional exercise or cognitive interventions.	"Individual Exercise Experimental Group (Standard Tai Chi Group): Traditional Tai Chi (Tai Ji Quan: Moving for Better Balance); Exercise Type: Focusing on postural alignment, weight transfer, balance recovery, and sensory integration, including core movements such as symmetrical movements and multi-directional stepping, without additional cognitive training;"	MoCA		
Lin 2024[47]	PC	MBE	Health education: once every 4 weeks, 60 minutes each time (45 minutes of lecture on cognitive protection knowledge + 15 minutes of Q&A), for a total of 12 weeks; maintain daily routine throughout, no additional exercise intervention.	Tai Chi (TC): 24-Form Tai Chi (mind-body exercise, including balance, posture control, and breathing regulation);<br>60 minutes per session (10 minutes warm-up + 40 minutes core exercises + 10 minutes cool-down review);<br>Instructed by a coach with over 20 years of teaching experience; some participants may practice at home an additional 1-3 times per week.	MoCA	WMS-MQ	
Lin 2025[17]	PC	AE MBE	"Routine medical care + activities of daily living: ward ventilation, dietary guidance, no additional exercise or cognitive intervention;<br>Intervention period: 24 weeks in total (12 weeks of intervention + 12 weeks of follow-up)"	1. Tai Chi Group (Individual Exercise): 8-Form Simplified Tai Chi (including the starting posture, Wild Horse Parts Mane, etc., totaling 10 movements), focusing on posture coordination and weight transfer, with no additional cognitive components;<br>2. Walking Group (Individual Exercise): Walking on flat ground + warm-up/cool-down stretching, with no additional cognitive components;<br>Implementation Method: Both groups are conducted within the facility. The Tai Chi group	MoCA	AVLT-Delayed	

Study	Control category	Exercise category	Control description	Intervention description	Global cognition outcome included in NMA	Memory outcome included in NMA	Executive -function outcomes included in NMA
**Liu 2021[48]**	PC	AE MBE	**Health Education**	**participates in group training, while the walking group participates in individual training, both with professional guidance.<br>Intervention Group 1 (Baduanjin Group): 60 minutes/session (15 minutes warm-up + 40 minutes core training + 5 minutes cool-down), 3 times/week, for a total of 6 months.<br>Exercise Type: Baduanjin Qigong (following the standards of the General Administration of Sport of China's "Health Qigong - Baduanjin", containing 10 movements);<br>Intervention Group 2 (Brisk Walking Group): 60 minutes/session (same as above), 3 times/week, for a total of 6 months.<br>Exercise Type: Aerobic brisk walking training.**	MoCA		
**Liu 2022[49]**	PC	MBE	**Routine Activity Control:<br>1. Maintaining only daily physical activities;<br>2. No additional exercise, cognitive training, or intervention;<br>3. Intervention duration: 12 weeks**	**Two separate exercise groups (without other intervention components):<br>1. EXER-TC Group: Motion-sensing game Tai Chi (based on Kinect device, mimicking a virtual instructor to complete Yang-style Tai Chi movements, including weight transfer and squats);<br>2. TC Group: Traditional Yang-style 24-form Tai Chi (simplified version, including warm-up, Tai Chi instruction, and relaxation);<br>Both groups underwent pure exercise intervention, without any cognitive training, medication, or other contaminants.**	MoCA		TMT-A;<br>TMT-B;<br>Delta-TMT
**Lü 2016[50]**	PC	MCE	**Maintain your existing lifestyle and do not participate in any new sports or cognitive training.**	**Momentum-based dumbbell training (compound resistance + coordination training): Using specially designed dumbbells with built-in eccentric pendulums, perform multi-directional rotational movements (clockwise/counterclockwise, forward/backward/left/right), including upper limb and full-body coordination training.**	ADAS-Cog		
**Mollinedo 2019[51]**	PC	RE DE	**1. Not engaging in any specific physical exercise;<br>2. Participate in regular activities in the nursing home (manual work, reading comprehension, cognitive**	**1. TG Group (Strength Training):<br>- Focuses on lower limb muscle groups, using resistance bands (TheraBands®) for resistance training;<br>- 60 minutes per session (10-minute warm-up + 45-**	MMSE		

Study	Control category	Exercise category	Control description	Intervention description	Global cognition outcome included in NMA	Memory outcome included in NMA	Executive -function outcomes included in NMA
			**stimulation), 2 times a week, 60 minutes each time**	**minute core training + 5-minute cool-down), with progressive intensity (from 2 sets of 10 reps to 4 sets of 15 reps);<br>2. MG Group (Multi-Set Aerobics Training):<br>- Primarily seated exercises, with standing exercises as an aid, including one fun aerobics game per week;<br>- 60 minutes per session, twice a week (1 basic aerobics session + 1 fun game session), balancing flexibility and coordination training.**			
**Montero Odasso 2023[52]**	AC	ARE	**Active Control (Non-Exercise Intervention Comparison, Balance-Stretching Training):<br>1. Intervention Type: Stretching, balance, toning training (no intensity/volume progress);<br>2. Session Duration: 60 minutes (90 minutes total session, including 30 minutes of sham cognitive training);<br>3. Frequency: 3 times/week;<br>4. Intervention Duration: 20 weeks; No cognitive training or vitamin D intervention**	**Resistance plus aerobic**	MoCA	Delayed Recall	TMT-A；TMT-B；DSST；DST-F；DST-B
**Nascimento 2014[53]**	PC	MCE	**No-Movement Control (Sedation Only):<br>1. Intervention Content: No participation in any regular exercise (at least 1 year of history of no regular exercise, maintaining a sedentary state during the intervention period);<br>2. No cognitive training or other interventions, only maintenance of daily activities.**	**1. Core components: Aerobic training (walking, jogging) + Resistance training (resistance bands, barbells) + Balance/coordination training (obstacle walking, vestibular stimulation);<br>2. Divided into 4 progressive stages, including a complete warm-up, main exercise, and cool-down.**	MoCA		
**Pieruccini Faria 2025[13]**	AC	ARE	**Balance + Stretch Training: Includes balance, stretching and shaping movements, no intensity/volume increments; 90 minutes each time (30**	**Core Intervention: Combined Aerobic-Resistance Exercise (based on exercise guidelines for older adults, standardized procedure + progressive design);**	MoCA		

Study	Control category	Exercise category	Control description	Intervention description	Global cognition outcome included in NMA	Memory outcome included in NMA	Executive -function outcomes included in NMA
			minutes of fake cognitive training + 60 minutes of training), 3 times/week, fully supervised	Group Differences:<br>1. Ex+Cog+VitD: Combined cognitive training (Neuropeak software, focusing on working memory/attention) + Vitamin D (10,000 IU/session, 3 times/week);<br>2. Ex+Cog: Combined cognitive training + placebo;<br>3. Ex+VitD: Combined sham cognitive training + Vitamin D;<br>4. Ex: Combined sham cognitive training + placebo;<br>Cognitive Training/Sham Training: 30 minutes/session, administered via tablet device.			
Qi 2019[54]	PC	DE	They receive only routine care, without any exercise interventions or cognitive-impairing medications (such as donepezil or memantine).	Individual exercise intervention: Specialized moderate-intensity dance aerobics (SDMIAD) containing 8 types of core movements (knee bending, calf raises, punching, shoulder movements, leg kicks, etc.). It requires memorizing the movements and concentrating attention, and is a compound exercise combining "aerobic exercise and cognitive coordination".	MoCA	WMS-LMII	
Rivas Campo 2023[55]	PC	MCE	1. General physical activity recommendations (based on the Pan American Health Organization PAHO guidelines);<br>2. Craft activities (drawing mandalas, decorating picture frames);<br>3. Frequency: 3 times/week, 45 minutes each time, for 12 weeks;<br>4. Instructed by an occupational therapist, assisted by a nursing assistant.	High-Intensity Functional Training (HIFT) is a variant of multi-joint functional interval training (HIIT).	MoCA		TMT-A; TMT-B; VFT; DSST; d2-CON
Rojasavastera 2020[56]	PC	AE	1. No training interventions will be provided;<br>2. Dementia-related health education will be provided during screening;<br>3. Daily living activities will be maintained, with follow-up periods consistent with the intervention group.	1. AOGT Group (Motion Observation + Gait Training):<br>- 65 minutes per session (5 minutes motion observation + 5 minutes warm-up + 40 minutes gait training + 5 minutes cool-down + 10 minutes stretching);<br>- Motion observation: Watching videos of healthy individuals walking normally (metronome 120	MoCA		

Study	Control category	Exercise category	Control description	Intervention description	Global cognition outcome included in NMA	Memory outcome included in NMA	Executive -function outcomes included in NMA
				beats/minute, stride 60cm);<br>- Gait training: Initially assisted by a metronome and ground markers, later progressing to independent walking without assistance;<br>2. GT Group (Gait Training Only):<br>- Intervention duration and structure are the same as the AOGT group, except the motion observation segment is replaced with watching Van Gogh's abstract paintings (to avoid emotional interference);<br>3. Both groups first complete 12 in-house training sessions (2-3 times per week), followed by one month of home-based self-training (3 times per week)			
Silva 2025[8]	PC	AE RE	Blank control group (does not participate in any training intervention, maintains daily routine, and only completes baseline and post-test assessments)	1. ST: Strength training only (hip and knee-dominant movements, upper and lower limb pushing and pulling, core training, with progressively increasing load); 2. STCT: Strength training + cognitive training (Fit4Alz software, focusing on memory, attention, and executive function, 20 minutes/session, performed after workout); 3. AT: Aerobic training only (including jump jacks, squats, lunges, etc., divided into Plan A/B, 3 variations); 4. ATCT: Aerobic training + cognitive training (same cognitive training program as STCT).	MoCA		
Song 2019[57]	PC	AE	Health Education Control Group (Attention Placebo):<br>1. Intervention Content: 8 bi-weekly health classes (45 minutes/session), excluding exercise and brain health-related content;<br>2. Only general health advice is provided, without cognitive or exercise interventions;<br>3. Intervention Duration: 16 weeks"	Single-intensity exercise intervention (no other intervention components):<br>1. Exercise type: Moderate-intensity aerobic stepping exercise (core activity is stepping up and down on a 10cm high step stool, combined with daily upper limb movements);<br>2. No cognitive training, medication, or other interventions; this is a pure aerobic exercise intervention."	MoCA		
Song 2023[58]	PC	DE	Health education (excluding exercise-related content): Group education sessions will be held once every two	Simple aerobic dance: a structured group exercise combined with music, including stepping up and down on a step stool and upper limb movements	MoCA		

Study	Control category	Exercise category	Control description	Intervention description	Global cognition outcome included in NMA	Memory outcome included in NMA	Executive -function outcomes included in NMA
			weeks, for a total of 8 sessions, each lasting 45 minutes, covering topics such as chronic disease management, healthy lifestyles, and insomnia management.	that simulate daily activities, mainly at a moderate intensity.			
Suzuki 2012[59]	PC	MCE	1. Health promotion education intervention: Attend 3 educational sessions within 12 months, covering topics such as aging, healthy eating, oral care, brain imaging diagnosis, urinary incontinence prevention, and health checkups;<br>2. No exercise or cognitive-related interventions: No exercise guidance or cognitive training will be provided.	Multi-component exercise (hybrid exercise mode):<br>1. Core content: Aerobic training (step training, endurance walking, outdoor walking), strength training, balance training, dual-task training (integrating cognitive tasks into exercise, such as composing poems while walking, memorizing step patterns);<br>2. Each session lasts 90 minutes (10 minutes warm-up + 20 minutes strength training + 60 minutes aerobic/balance/dual-task training);<br>3. Conducted in groups (16-17 people per group), combined with home exercise (independent exercise + outdoor walking), using a pedometer and notebook to record duration/steps, self-monitoring to promote adherence.	MMSE	WMS-LMII	DSC；<br>LVFT；<br>CVFT；<br>SCWTI；<br>SCWT II
Suzuki 2013[60]	PC	MCE	1. Health promotion education intervention: Participation in 2 educational sessions covering topics such as healthy eating, oral care, urinary incontinence prevention, and health check-ups;<br>2. No exercise or cognitive-related interventions; no exercise guidance provided.	Multi-component exercise (hybrid exercise mode):<br>1. Core content: Aerobic training (step training, endurance walking, outdoor walking), strength training, balance training, dual-task training (integrating cognitive tasks into exercise, such as composing poetry while walking);<br>2. Each session lasts 90 minutes (10 minutes of warm-up and stretching + 20 minutes of strength training + 60 minutes of aerobic/balance/dual-task training);<br>3. Conducted in groups (16-17 people per group), combined with home exercises (strength training + walking), using a pedometer and manual for self-monitoring.	MMSE	WMS-LMII	
Tao 2019[61]	PC	AE MBE	1. Maintain existing lifestyle without specific exercise intervention;<br>2. Receive a health education lecture once every 8 weeks, 30 minutes each time, on MCI prevention-related	1. Baduanjin (Eight Pieces of Brocade) Set (Mind-Body Exercise):<br>- Based on the 2003 National Sports General Administration's "Standard for Baduanjin Qigong," containing 10 movements;	MoCA		

Study	Control category	Exercise category	Control description	Intervention description	Global cognition outcome included in NMA	Memory outcome included in NMA	Executive -function outcomes included in NMA
			knowledge.	- 60 minutes per session (15 minutes warm-up + 40 minutes core training + 5 minutes cool-down);<br>2. Brisk Walking Set (Aerobic Exercise):<br>- Community-based brisk walking training;<br>- 60 minutes per session (15 minutes warm-up + 40 minutes brisk walking + 5 minutes cool-down)			
Tomoto 2021[62]	AC	AE	Active Control (No Aerobic Component):<br>1. Intervention Content: Upper and lower limb stretching exercises, later supplemented with low-resistance TheraBand training;<br>2. Intensity: Heart rate below 50% of maximum heart rate;<br>3. Frequency and duration consistent with the experimental group, except for the absence of aerobic metabolic stimulation.	Individual Exercise Experimental Group (No Cognitive/Drug Confounding):<br>1. Exercise Type: Moderate to high intensity aerobic training, primarily brisk walking (treadmill/outdoor);	MMSE		
Uysal 2023[63]	-	RE ARE	Single-exercise intervention (lower limb strength training):<br>1. Lower limb strength training only (chair squats, hip flexion and extension, etc.);<br>2. No aerobic or dual-task training components;<br>Intervention period: 12 weeks, consistent with the experimental group.	Individual Exercise Experiment Group 1 (AG): Aerobic + Lower Limb Strength Training (No Cognitive Confounding);<br>Exercise Format: Treadmill aerobic (5 warm-up + 20 loads + 5 cool-down) + Lower Limb Strength Training (chair squats, etc.);<br>Individual Exercise Experiment Group 2 (DG): Dual-Task + Lower Limb Strength Training (No Cognitive Confounding);<br>Exercise Format: Balance training + Cognitive tasks (color naming, simple arithmetic, etc.) + Lower Limb Strength Training;<br>Individual Exercise Experiment Group 3 (ADG): Aerobic + Dual-Task + Lower Limb Strength Training (No Cognitive Confounding);<br>Exercise Format: Combining all exercise content from AG and DG;<br>Implementation Method: All sessions were led by experienced physical therapists, with individual group training including rest intervals.	MMSE		
Varela	AC	AE	Recreational activities (without	Moderate to low intensity aerobic	MMSE		

Study	Control category	Exercise category	Control description	Intervention description	Global cognition outcome included in NMA	Memory outcome included in NMA	Executive -function outcomes included in NMA
2012[64]			physical activity): playing cards, reading newspapers, crafts, etc., depending on the frequency of participation.				
Wang 2020[65]	PC	MCE	Participate in health promotion courses (covering topics such as cognitive impairment and healthy eating), without engaging in any exercise interventions.	Structured physical exercises (compound exercises, including upper and lower limb training): 10-minute warm-up + 40-minute core training (hopscotch, sandbag throwing, etc.) + 10-minute cool-down; supervised for the first 12 weeks, followed by 12 weeks of independent practice at home.	MoCA		
Wang 2024[66]	PC	DE	Maintain existing lifestyle habits and do not participate in additional physical exercise.	Chinese Square Dance (Aerobic Group Exercise): Adopts the 17th set of "Chinese Dream Team Happy Dance Steps" fitness exercises, including upper and lower limb, shoulder, chest, waist and abdomen movements. The movements are simple and repetitive, and are coordinated with the rhythm of the music.	MoCA		SCWT III；N-back Task；more-odd shifting task
Wei 2014[67]	PC	MCE	Maintain existing lifestyle and recreational activities (such as playing cards), and do not accept any specific sports interventions.	Handball-related multi-component training: 30 minutes/time (5 minutes warm-up + 25 minutes core training), 5 times/week, a total of 6 months. Types of sports: including throwing and catching training, hitting training, bouncing passing, circle passing, grabbing training, etc. 8 categories, focusing on compound sports of hand-eye coordination, agility, and body control; Training progress: 2 training sessions per day in the first 2 weeks, 4 training sessions per day thereafter (progressive difficulty)	MMSE		
Wu H 2025[68]	PC	DE	Health education only (including prevention of dementia risk factors, Mediterranean diet, healthy lifestyle, and insomnia management), without aerobic exercise intervention.	Simple aerobic dance: a structured dance that combines music and physical movement, including coordinated upper and lower limb exercises.	MoCA	WMS-LMII	
Wu TT 2025[69]	AC	RE	Standard Senior Fitness Exercises (compiled by Shanghai University of Sport): Includes finger exercises, clapping exercises, and stretching exercises; group training for the first	Resistance Training (RT): A hybrid online-offline model, with the first 6 weeks of offline group training followed by 6 weeks of online real-time guidance via Tencent Meeting; using resistance bands to train muscle groups including shoulders,	MoCA	AVLT-Delayed	

Study	Control category	Exercise category	Control description	Intervention description	Global cognition outcome included in NMA	Memory outcome included in NMA	Executive -function outcomes included in NMA
			6 weeks, followed by home training for the next 6 weeks; 3 times a week, 40 minutes each time (5 minutes warm-up + 30 minutes exercise + 5 minutes cool-down + 10 minutes health education); intensity: Borg Self-Reflective Fatigue Scale score 10-14.	back, chest, upper and lower limbs, glutes, thighs, and calves; progressing in three phases: initial (weeks 1-6), intermediate (weeks 7-10), and final (weeks 11-12).			
Xia 2023[70]	PC	AE MBE	General comparison:<br>1. Intervention content: maintain the original lifestyle and do not impose specific exercise intervention;<br>2. Additional measures: attend 1 health lecture (nutrition, elderly care, etc.) every 8 weeks, 3 times in total; No exercise or cognitive training components	1. Baduanjin Group: A Traditional Chinese Medicine mind-body exercise based on the "Health Qigong - Baduanjin" standard, consisting of 8 core movements plus preparatory/closing postures, combined with breathing regulation and meditation;<br>2. Brisk Walking Group: Aerobic training, brisk walking on flat ground, without additional cognitive intervention;<br>Both groups receive independent interventions, with separate training areas to avoid cross-interference.	MoCA		
Yang 2022[71]	PC	ARE	Health Lecture Comparison:<br>1. Intervention Content: 8 health-related topic lectures (nutrition, exercise recommendations for geriatric disease prevention);<br>2. Each lecture lasts 30 minutes, with no exercise or cognitive training components;<br>3. Intervention Period: Synchronized with the exercise group (12 weeks)	Single-Action Experimental Group (No Other Interventions):<br>1. Exercise Type: Combined exercise (aerobic training + progressive resistance training);<br>2. Aerobic Training: Indoor/outdoor walking, jumping jacks, box walking;<br>3. Resistance Training: Targeting 6 major muscle groups (hamstrings, quadriceps, etc.), using free weights and resistance bands; No cognitive training, drugs, or other contaminants were used; this was a pure exercise intervention.	MMSE		TMT-A;<br>DSST
Yoon 2016[72]	AC	RE	Maintain daily activities, with one hour of static/dynamic stretching training once a week, without additional exercise or cognitive training.	1. HSPT Group: High-speed power resistance training with elastic bands (rapid concentric contraction + 1-second pause + >2-second eccentric contraction); 2. LSST Group: Low-speed strength resistance training with elastic bands (>2-second concentric contraction + 1-second pause + >2-second eccentric contraction); Both include multi-muscle group training of the upper and lower	MMSE		

Study	Control category	Exercise category	Control description	Intervention description	Global cognition outcome included in NMA	Memory outcome included in NMA	Executive -function outcomes included in NMA
Yu AP 2022[73]	-	MBE MCE	**No intervention: Maintain daily activities without providing any exercise or cognitive training intervention; Intervention period: 24 weeks.**	**limbs.<br>1. Tai Chi Group (TC, Individual Exercise): 24-Form Yang Style Tai Chi, including meditation elements, no additional cognitive training;<br>2. Regular Exercise Group (EX, Individual Exercise): Compound regular exercise (10 minutes warm-up and stretching + 20 minutes strength training + 20 minutes aerobic exercise + 10 minutes cool-down and stretching), without meditation or cognitive components;<br>Implementation Method: Professional coaching (Tai Chi coach is certified, regular exercise coach is certified fitness coach), some sessions during the pandemic will be video-based.**	MoCA-HK	Delayed Recall	
Yu DJ 2022[74]	AC	AE	**1. Individual stretching exercises once a week, 75 minutes each time (covering major muscle groups such as chest, back, and lower limbs);<br>2. Maintain existing lifestyle habits, without other specific exercise interventions.**	**All activities are walking (aerobic exercise), divided into 4 groups based on intensity and frequency:<br>1. Moderate Intensity (3.5 METs):<br><br>- M1: Once a week, 150 minutes each time (including 5 minutes warm-up + 140 minutes core walking + 5 minutes cool-down, including two 10-minute rest periods);<br>- M3: Three times a week, 50 minutes each time (including 5 minutes warm-up + 40 minutes core walking + 5 minutes cool-down);<br>2. High Intensity (7 METs):<br>- V1: Once a week, 75 minutes each time (including 5 minutes warm-up + 65 minutes core walking + 5 minutes cool-down, including one 10-minute rest period);<br>- V3: Three times a week, 25 minutes each time (including 5 minutes warm-up + 15 minutes core walking + 5 minutes cool-down);<br>3. All walking groups are performed on a treadmill, maintaining the target heart rate using a heart rate monitor.**	HK-MoCA		
Zhang 2023[75]	PC	AE	**Health Education Comparison:<br>1. Intervention Content: Attendance at 3 health knowledge lectures**	**Two separate exercise groups (without other intervention components):<br>1. TCE+RTG group: Traditional Chinese medicine**	MoCA		

Study	Control category	Exercise category	Control description	Intervention description	Global cognition outcome included in NMA	Memory outcome included in NMA	Executive -function outcomes included in NMA
			(elderly cognitive impairment-related diseases, memory care, chronic disease prevention, etc.);<br>2. No exercise intervention;<br>3. Intervention Duration: Synchronized with the exercise group (12 weeks)	exercise + rhythm training (including joint stretching, aerobic exercise, acupressure massage, relaxation breathing exercises, accompanied by music rhythm);<br>2. WG group: Walking exercise (3 km outdoor walk, including 10 minutes of warm-up and cool-down);<br>Both groups were purely exercise interventions, without any cognitive training, drugs, or other contaminants.			
Zheng 2021[76]	PC	AE MBE	1. Maintain the original daily physical activity habits without specific exercise intervention;<br>2. Receive cognitive-related health education once every 8 weeks, 30 minutes each time (content includes nutrition, elderly care, and cognitive decline)	1. Baduanjin (Eight Pieces of Brocade) Group (Traditional Chinese Medicine Mind-Body Exercise):<br>- Based on the 2003 National Sports General Administration's "Standard for Baduanjin Qigong," including preparatory, 8 core exercises, and closing movements;<br>- 60 minutes per session (15 minutes warm-up + 40 minutes core training + 5 minutes cool-down);<br>2. Brisk Walking Group (Aerobic Exercise):<br>- Community-based brisk walking training;<br>- 60 minutes per session (15 minutes warm-up + 40 minutes brisk walking + 5 minutes cool-down)	MoCA	WMS-MQ	
Zhou 2025[77]	PC	MBE	Routine care: Maintain existing daily activities, without participating in any structured exercise interventions; Requires weekly physical activity levels < 600 METs-min, monitored every 4 weeks using the International Physical Activity Questionnaire-.	Intervention Group 1 (TCG): Simple Eight-Form Tai Chi: 45-50 minutes/session, intervention period 12 weeks; Exercise type: Four sets of eight-form Tai Chi (identical content, different order), focusing on weight transfer, limb coordination, and synchronized breathing, without additional cognitive/music intervention;	MoCA	WMS-LM II	SCWT；TMT-A；CDT

Appendix 2. Supplementary figures for global cognitive function analyses

1.Figure_S1_Risk_of_bias_summary_global_cognition

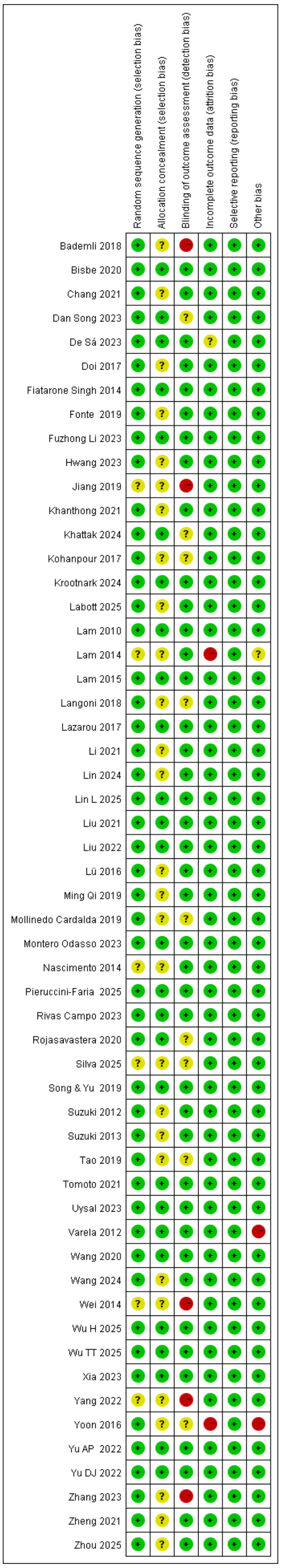

2.Figure_S2_Cumulative_ranking_probabilities_global_cognition

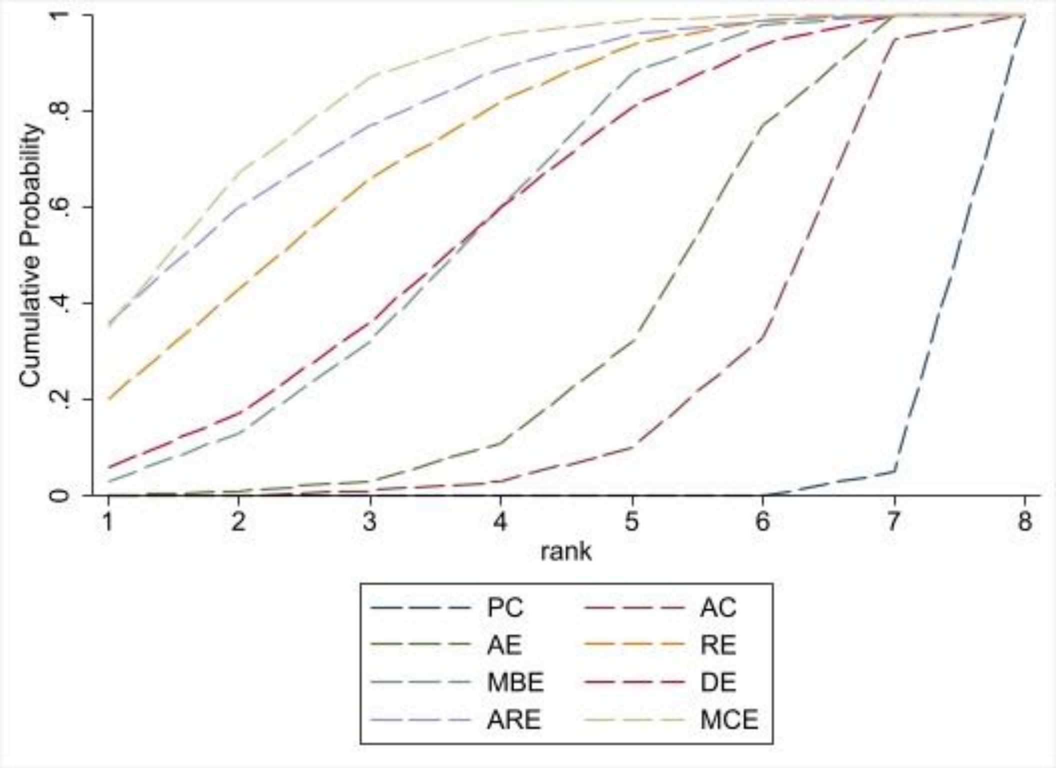

3.Figure_S3_Contribution_matrix_global_cognition

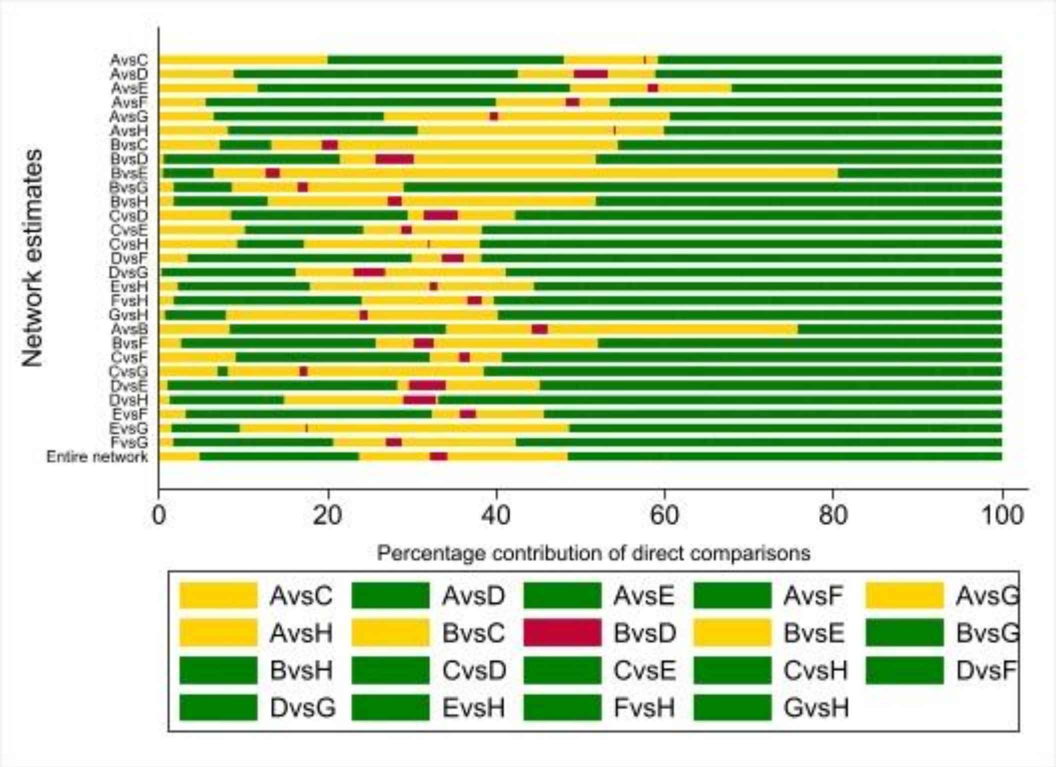

4.Figure_S4_Network_geometry_global_cognition

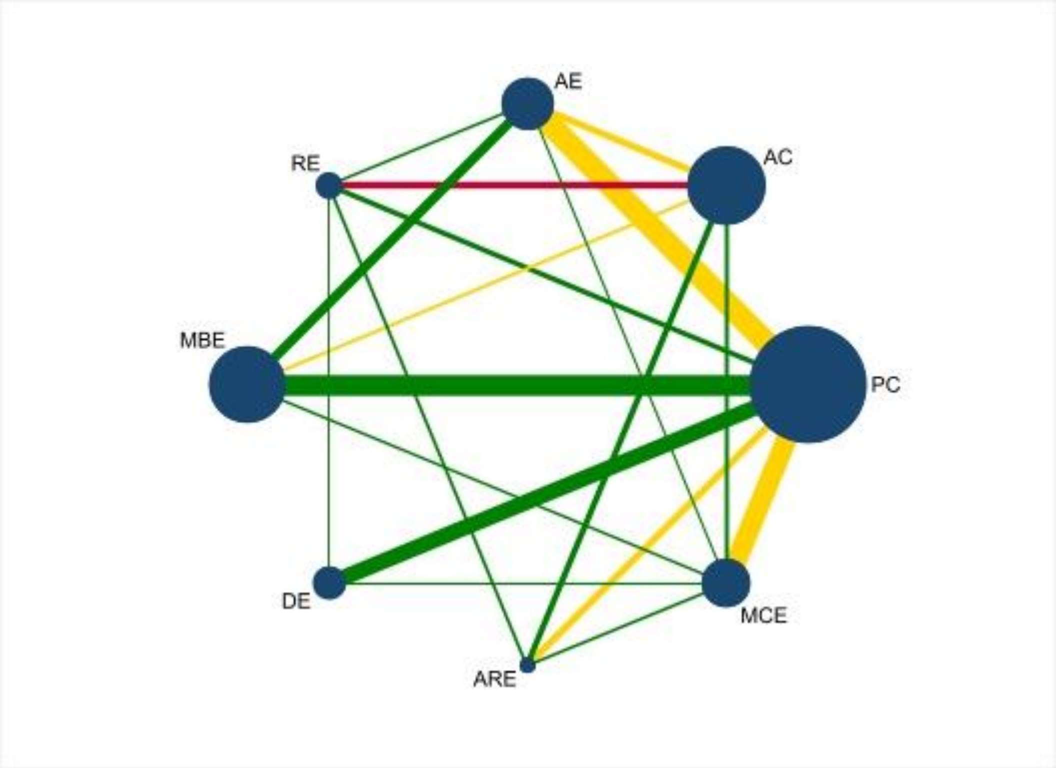

5.Figure_S5_Comparison_adjusted_funnel_plot_global_cognition

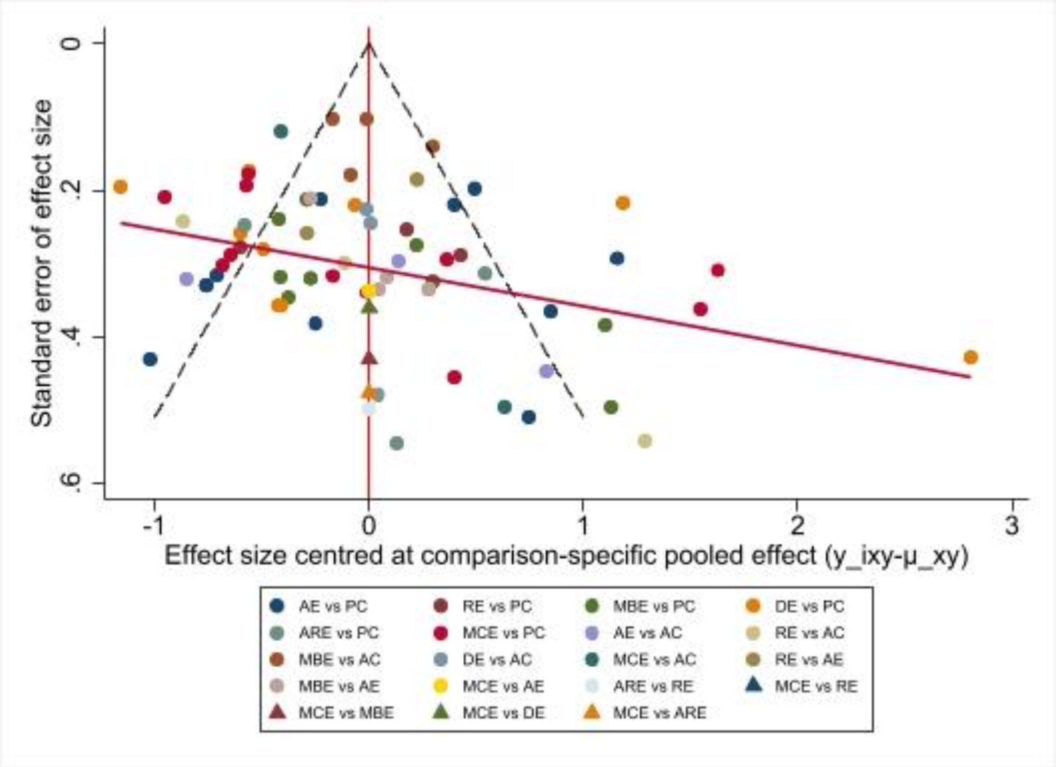

6.Figure_S6_League_table_global_cognition

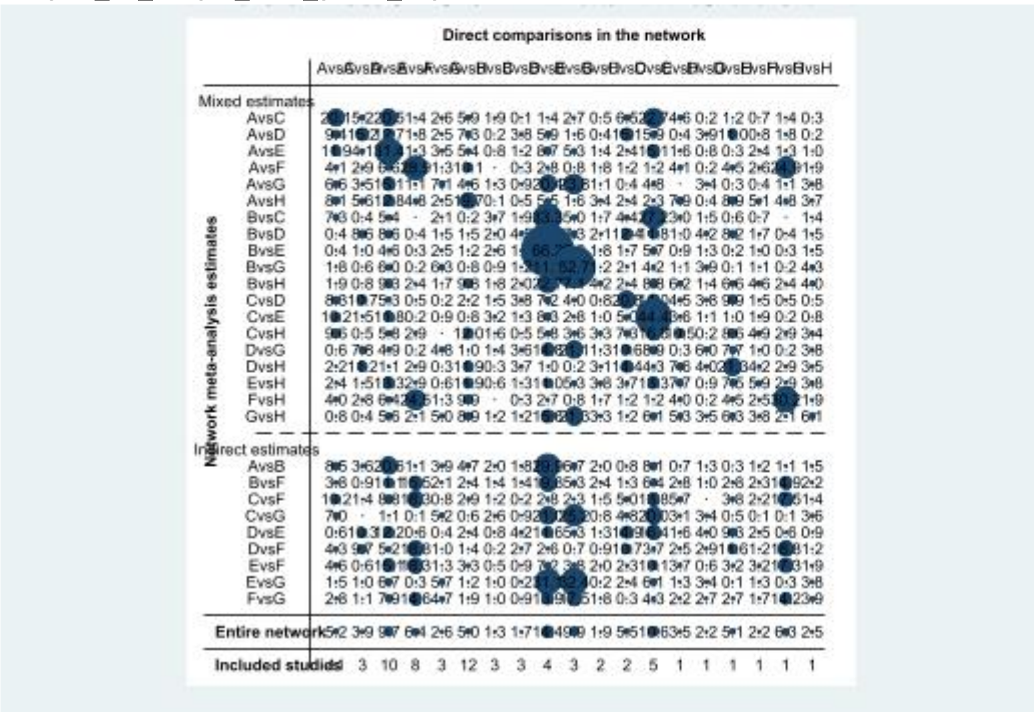

7.Figure_S7_SUCRA_values_global_cognition

Treatm~t	SUCRA	PrBest	MeanRank
PC	0.8	0.0	7.9
AC	20.2	0.0	6.6
AE	31.9	0.1	5.8
RE	71.8	20.4	3.0
MBE	56.4	3.2	4.1
DE	56.0	5.5	4.1
ARE	79.6	36.3	2.4
MCE	83.3	34.6	2.2

8.Figure_S8_Pairwise_forest_plot_global_cognition

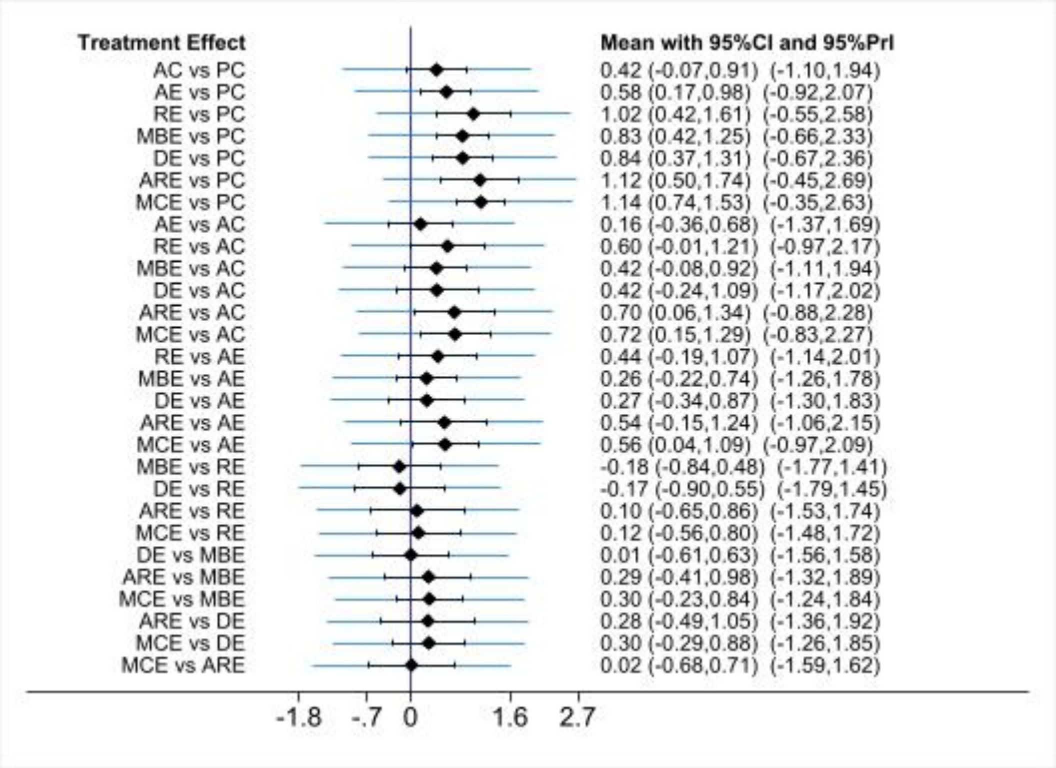

Appendix 3

1 Global cognition function

1.1 The loop-specific approach

Loop	IF	seIF	z_value	p_value	CI_95	Loop_Heterog_tau2
**RE-DE-ARE-MCE**	**2.558**	**0.83**	**3.08**	**0.002**	**(0.93,4.19)**	**0**
**AC-RE-ARE**	**2.196**	**1.004**	**2.188**	**0.029**	**(0.23,4.16)**	**0.245**
**PC-RE-ARE**	**2.133**	**0.868**	**2.457**	**0.014**	**(0.43,3.83)**	**0.262**
**AE-RE-DE-MCE**	**1.922**	**0.592**	**3.245**	**0.001**	**(0.76,3.08)**	**0**
**AC-AE-MCE**	**1.843**	**1.072**	**1.719**	**0.086**	**(0.00,3.94)**	**0.477**
**AE-MBE-MCE**	**1.824**	**0.562**	**3.244**	**0.001**	**(0.72,2.93)**	**0**
**PC-AE-MCE**	**1.302**	**0.901**	**1.446**	**0.148**	**(0.00,3.07)**	**0.488**
**AC-ARE-MCE**	**0.937**	**0.518**	**1.81**	**0.07**	**(0.00,1.95)**	**0**
**PC-MBE-MCE**	**0.661**	**0.902**	**0.732**	**0.464**	**(0.00,2.43)**	**0.404**
**PC-AE-RE**	**0.651**	**0.584**	**1.113**	**0.266**	**(0.00,1.80)**	**0.262**
**AE-RE-ARE-MCE**	**0.636**	**0.782**	**0.813**	**0.416**	**(0.00,2.17)**	**0**
**PC-AE-MBE**	**0.588**	**0.378**	**1.557**	**0.119**	**(0.00,1.33)**	**0.151**
**PC-AC-RE-MCE**	**0.519**	**0.967**	**0.537**	**0.591**	**(0.00,2.41)**	**0.526**
**AC-AE-MBE**	**0.467**	**0.353**	**1.325**	**0.185**	**(0.00,1.16)**	**0.047**
**AC-AE-RE**	**0.434**	**0.897**	**0.484**	**0.629**	**(0.00,2.19)**	**0.409**

**PC-DE-MCE**	**0.349**	**1.149**	**0.304**	**0.761**	**(0.00,2.60)**	**0.729**
**AC-RE-DE-MCE**	**0.296**	**1.589**	**0.186**	**0.852**	**(0.00,3.41)**	**0.598**
**AC-MBE-MCE**	**0.225**	**0.621**	**0.363**	**0.716**	**(0.00,1.44)**	**0.038**
**PC-ARE-MCE**	**0.215**	**1.078**	**0.2**	**0.842**	**(0.00,2.33)**	**0.552**
**PC-AC-MBE-ARE**	**0.152**	**0.446**	**0.34**	**0.734**	**(0.00,1.03)**	**0.07**
**PC-AC-AE-ARE**	**0.148**	**0.731**	**0.202**	**0.84**	**(0.00,1.58)**	**0.33**
**PC-AC-RE-MBE**	**0.098**	**0.542**	**0.18**	**0.857**	**(0.00,1.16)**	**0.109**
**PC-RE-DE**	**0.05**	**1.368**	**0.037**	**0.971**	**(0.00,2.73)**	**0.847**

1.2 The node-splitting method

**Side**	**Direct**		**Indirect**		**Difference**		
	Coef.	Std. Err.	Coef.	Std. Err.	Coef.	Std. Err.	P>z
**A C**	**0.6429229**	**0.2364095**	**0.3597373**	**0.4284962**	**0.2831856**	**0.4897623**	**0.563**
**A D**	**1.250526**	**0.4392368**	**0.7943925**	**0.4258311**	**0.4561331**	**0.6127751**	**0.457**
**A E**	**0.8226691**	**0.2484487**	**0.8700043**	**0.430298**	**-0.0473352**	**0.4972724**	**0.924**
**A F**	**0.8036088**	**0.2555486**	**1.157685**	**0.7264258**	**-0.3540761**	**0.7697757**	**0.646**
**A G**	**0.9130977**	**0.4633347**	**1.310786**	**0.4441523**	**-0.397688**	**0.6417808**	**0.535**
**A H**	**1.143144**	**0.2336628**	**1.122045**	**0.4097054**	**0.0210988**	**0.472109**	**0.964**
**B C**	**0.4218452**	**0.4589741**	**0.0288704**	**0.3244797**	**0.3929747**	**0.5620353**	**0.484**
**B D**	**1.012266**	**0.4593852**	**0.2455378**	**0.4234532**	**0.7667277**	**0.624974**	**0.22**
**B E**	**0.270674**	**0.363097**	**0.5662231**	**0.3649299**	**-0.2955491**	**0.514792**	**0.566**
**B G**	**0.5153074**	**0.4517845**	**0.9173519**	**0.4815232**	**-0.4020445**	**0.6605832**	**0.543**
**B H**	**0.3840331**	**0.5540093**	**0.8479807**	**0.3408352**	**-0.4639477**	**0.6501176**	**0.475**
**C D**	**0.1395697**	**0.5268233**	**0.619228**	**0.4094518**	**-0.4796583**	**0.6667082**	**0.472**
**C E**	**0.4189122**	**0.3466723**	**0.0937538**	**0.3495158**	**0.3251585**	**0.4922601**	**0.509**
**C H**	**1.802423**	**0.7680664**	**0.3947568**	**0.2792498**	**1.407666**	**0.8172554**	**0.085**
**D F**	**-0.325855**	**0.7712109**	**-0.125907**	**0.4269106**	**-0.199948**	**0.8815258**	**0.821**
**D G**	**1.772561**	**0.8460492**	**-0.3061598**	**0.4191788**	**2.078721**	**0.9441982**	**0.028**
**E H**	**-0.3452088**	**0.8298564**	**0.3828616**	**0.2910761**	**-0.7280704**	**0.8793524**	**0.408**
**F H**	**-0.0094752**	**0.7990174**	**0.3459349**	**0.3256562**	**-0.3554101**	**0.862833**	**0.68**
**G H**	**0.4486954**	**0.856089**	**-0.0737947**	**0.3932974**	**0.5224902**	**0.9423335**	**0.579**

2 Cognition-related memory function

2.1 The loop-specific approach

**Loop**	**IF**	**seIF**	**z_value**	**p_value**	**CI_95**	**Loop_Heterog_tau2**
**PC-AC-ARE-MCE**	**2.337**	**1.09**	**2.143**	**0.032**	**(0.20,4.47)**	**0.197**
**PC-AC-RE-ARE**	**1.886**	**0.67**	**2.814**	**0.005**	**(0.57,3.20)**	**0**

**PC-AC-MBE-ARE**	**1.517**	**0.94**	**1.614**	**0.107**	**(0.00,3.36)**	**0.084**
**AC-AE-RE-MBE**	**1.276**	**0.523**	**2.439**	**0.015**	**(0.25,2.30)**	**0.02**
**PC-AE-RE**	**0.746**	**0.436**	**1.713**	**0.087**	**(0.00,1.60)**	**0**
**PC-MBE-MCE**	**0.562**	**0.857**	**0.656**	**0.512**	**(0.00,2.24)**	**0.263**
**PC-AE-MBE**	**0.503**	**0.564**	**0.891**	**0.373**	**(0.00,1.61)**	**0.168**
**PC-AC-RE-MCE**	**0.451**	**0.744**	**0.606**	**0.545**	**(0.00,1.91)**	**0.111**
**PC-AC-RE-MBE**	**0.381**	**0.638**	**0.598**	**0.55**	**(0.00,1.63)**	**0.074**
**AC-MBE-MCE**	**0.281**	**0.475**	**0.591**	**0.555**	**(0.00,1.21)**	**0**
**PC-DE-MCE**	**0.134**	**0.639**	**0.209**	**0.834**	**(0.00,1.39)**	**0.096**

2.2 The node-splitting method

**Side**	**Direct**		**Indirect**		**Difference**		
	Coef.	Std. Err.	Coef.	Std. Err.	Coef.	Std. Err.	P>z
**A C ***	**0.427247**	**0.307207**	**0.112287**	**0.782949**	**0.314961**	**0.843289**	**0.709**
**A D**	**0.707782**	**0.515525**	**0.794476**	**0.453305**	**-0.08669**	**0.687241**	**0.9**
**A E**	**0.873764**	**0.246162**	**0.683254**	**0.468183**	**0.190511**	**0.52672**	**0.718**
**A F**	**0.680792**	**0.261048**	**0.75362**	**0.631003**	**-0.07283**	**0.682405**	**0.915**
**A G**	**0.981216**	**0.648082**	**-0.87985**	**0.489783**	**1.861071**	**0.81234**	**0.022**
**A H**	**0.327939**	**0.306274**	**0.882224**	**0.392083**	**-0.55428**	**0.497678**	**0.265**
**B D**	**0.371593**	**0.355995**	**-0.29504**	**0.536286**	**0.666628**	**0.643951**	**0.301**
**B E**	**0.105549**	**0.312349**	**0.478502**	**0.411063**	**-0.37295**	**0.516294**	**0.47**
**B G**	**-1.27205**	**0.422001**	**0.589018**	**0.694126**	**-1.86107**	**0.81234**	**0.022**
**B H**	**0.41675**	**0.441508**	**-0.38319**	**0.379505**	**0.799937**	**0.582197**	**0.169**
**C D**	**-0.1877**	**0.465157**	**1.017423**	**0.510132**	**-1.20512**	**0.690613**	**0.081**
**C E**	**0.842928**	**0.353118**	**-0.18572**	**0.431516**	**1.028648**	**0.559357**	**0.066**
**E H**	**0.04714**	**0.60866**	**-0.3916**	**0.332776**	**0.438739**	**0.693944**	**0.527**
**F H**	**-0.20116**	**0.568585**	**-0.12833**	**0.377344**	**-0.07283**	**0.682406**	**0.915**

2.3 Comparison adjusted funnel plot

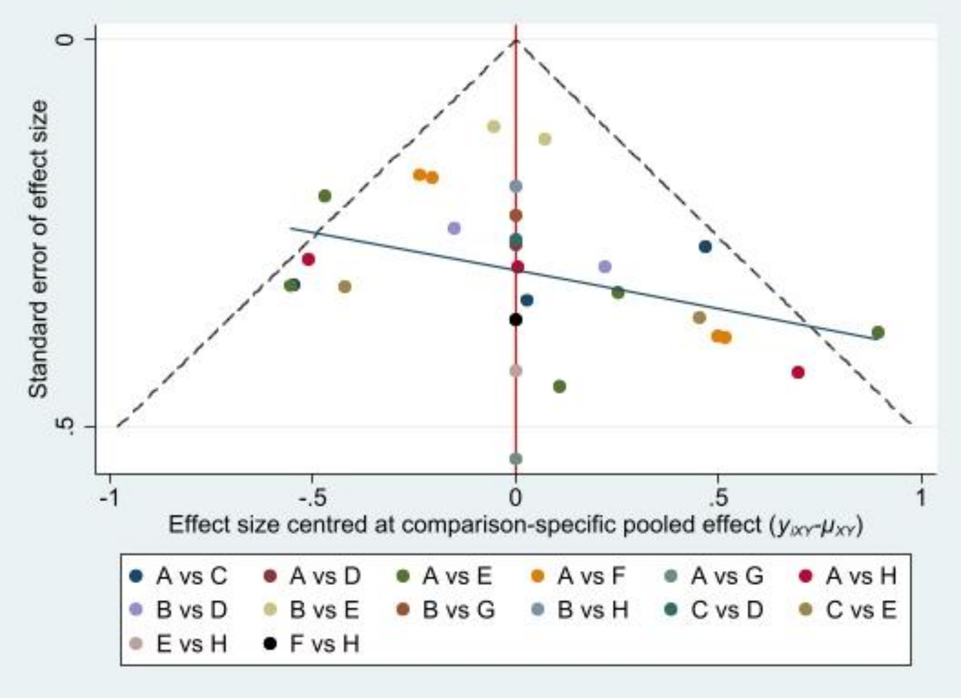

2.4 Pairwise forest plot

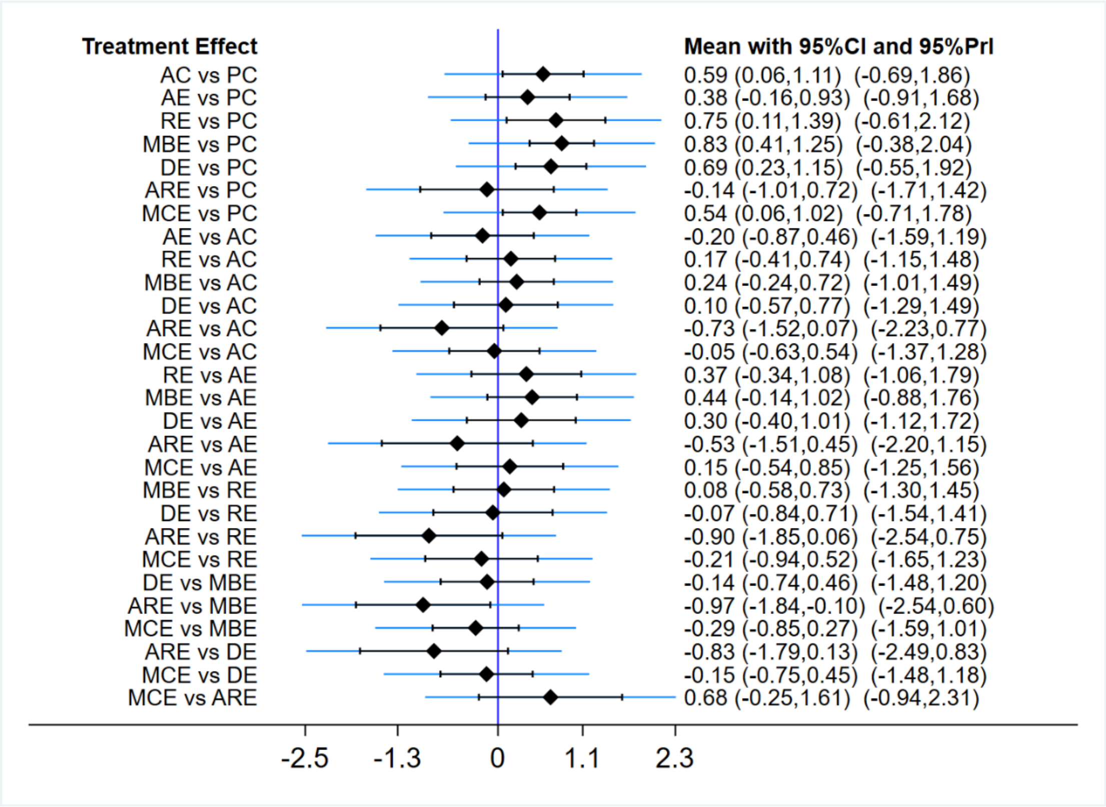

2.5 League table

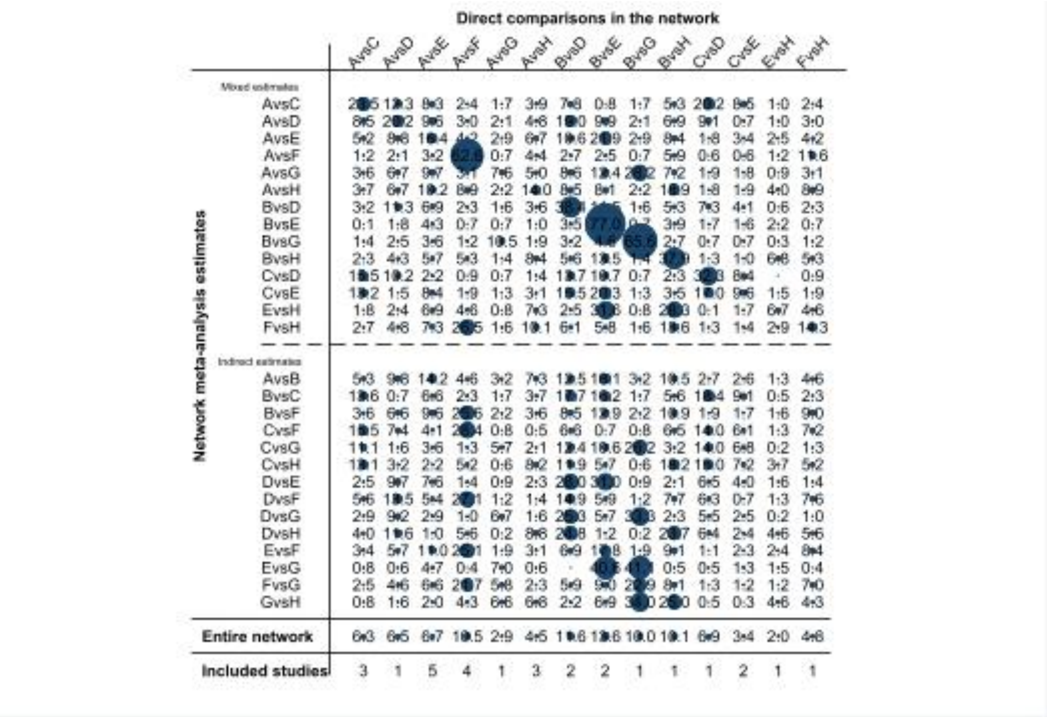

3 Cognition-related executive function

3.1 The loop-specific approach

Loop	IF	seIF	z_value	p_value	CI_95	Loop_Heterog_tau2
**PC-AC-RE-ARE**	**1.129**	**1.301**	**0.868**	**0.385**	**(0.00,3.68)**	**0.566**
**PC-AC-DE-ARE**	**1.068**	**1.885**	**0.567**	**0.571**	**(0.00,4.76)**	**1.113**
**PC-MBE-ARE**	**0.223**	**2.024**	**0.11**	**0.912**	**(0.00,4.19)**	**1.901**
PC-AC-RE-DE	**0.124**	**0.958**	**0.129**	**0.897**	**(0.00,2.00)**	**0.225**

3.2 The node-splitting method

Side	Direct		Indirect		Difference		
	Coef.	Std. Err.	Coef.	Std. Err.	Coef.	Std. Err.	P>z
**A E**	**0.510229**	**0.530784**	**1.15679**	**1.164663**	**-0.64656**	**1.279937**	**0.613**
**A F**	**1.349673**	**0.6504475**	**0.9138913**	**1.095555**	**0.435782**	**1.27402**	**0.732**
**A G**	**1.090229**	**0.6930937**	**1.643825**	**1.159171**	**-0.5536**	**1.350576**	**0.682**
**A H**	**1.43734**	**0.6381179**	**0.9815766**	**0.80836**	**0.455763**	**1.030284**	**0.658**
**B D ***	**0.768925**	**0.8720827**	**-0.8860722**	**632.5859**	**1.654997**	**632.5864**	**0.998**
**B E**	**0.094675**	**0.8754887**	**-0.5518959**	**0.933686**	**0.646571**	**1.279941**	**0.613**
**B G**	**0.653507**	**0.9020674**	**0.0999025**	**1.005156**	**0.553605**	**1.350579**	**0.682**
**B H**	**-0.10421**	**0.8303723**	**1.058132**	**0.896457**	**-1.16234**	**1.221946**	**0.341**
**C H ***	**0.381275**	**0.8668273**	**2.13831**	**632.2275**	**-1.75703**	**632.2282**	**0.998**
**F H**	**0.262721**	**0.938256**	**-0.1730534**	**0.861864**	**0.435774**	**1.274023**	**0.732**

3.3 Comparison adjusted funnel plot

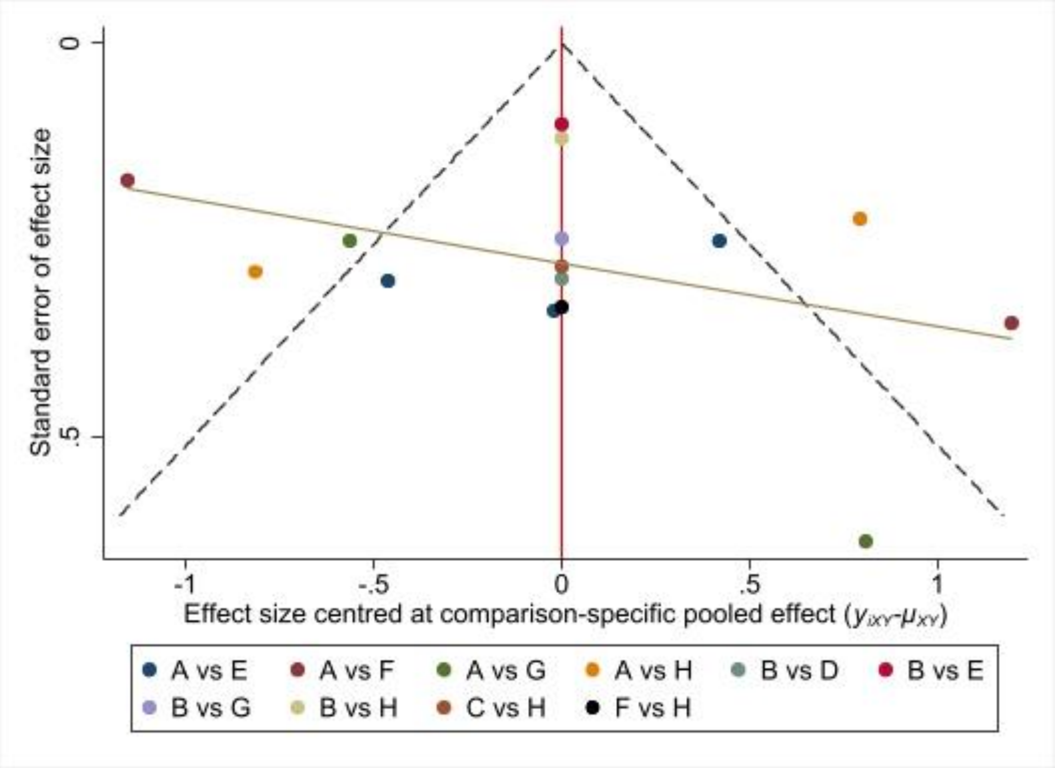

3.4 Pairwise forest plot

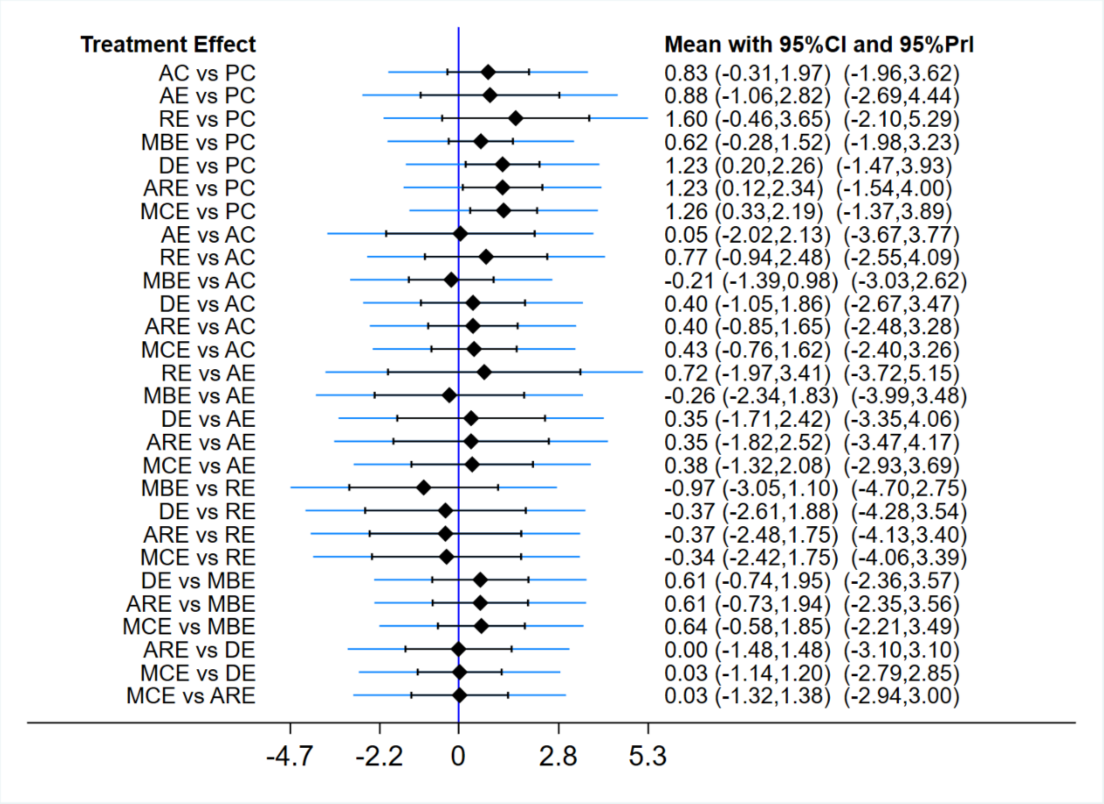

3.5 League table

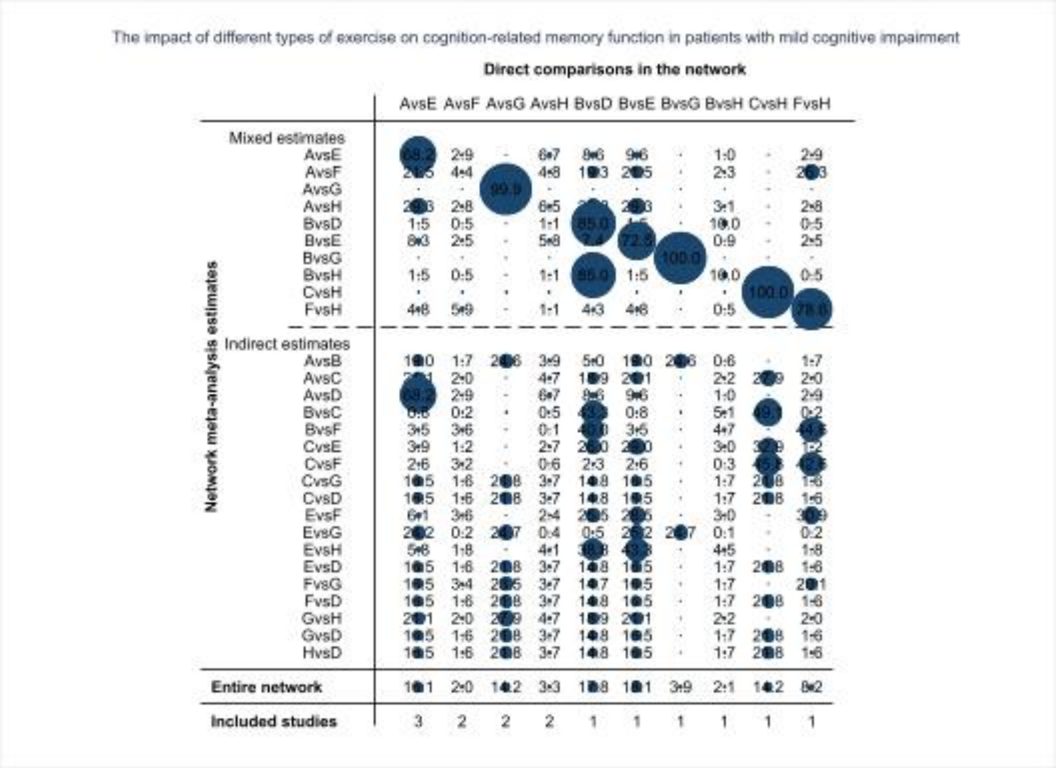

Appendix 4 sensitivity analysis results

1. Analysis results of r=0.5

Treatm~t	SUCRA	PrBest	MeanRank
PC	0.8	0.0	7.9
AC	20.2	0.0	6.6
AE	31.9	0.1	5.8
RE	71.8	20.4	3.0
MBE	56.4	3.2	4.1
DE	56.0	5.5	4.1
ARE	79.6	36.3	2.4
MCE	83.3	34.6	2.2

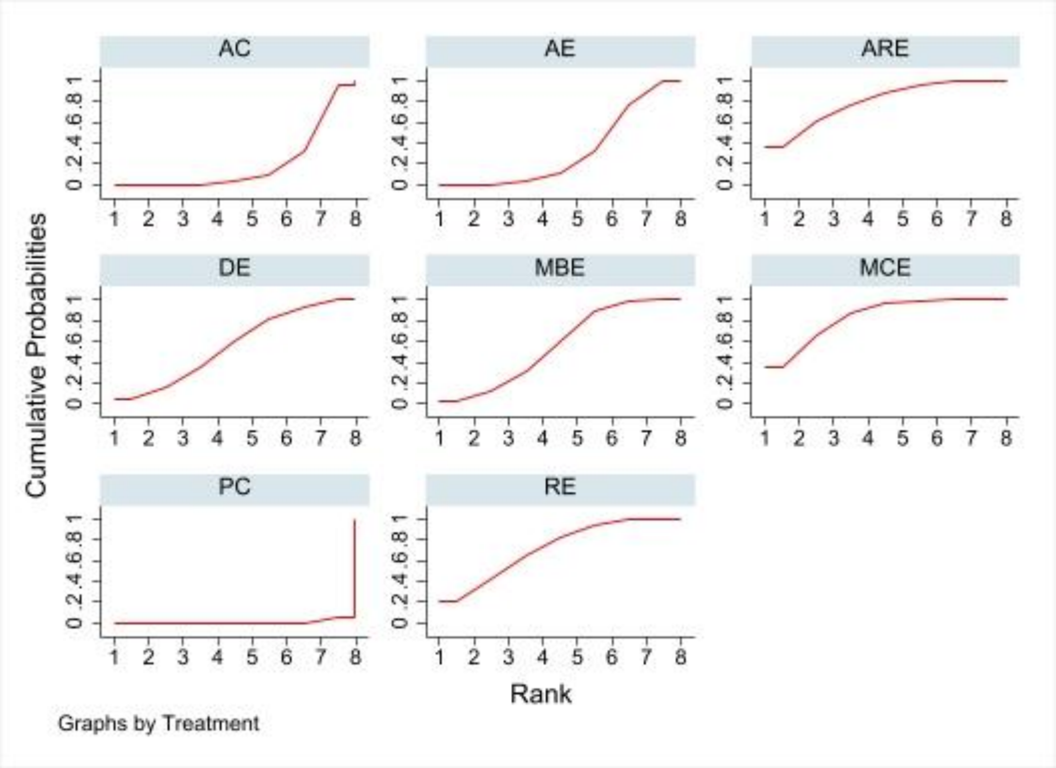

2. Analysis results of r=0.3

Treatm~t	SUCRA	PrBest	MeanRank
PC	0.9	0.0	7.9
AC	20.6	0.0	6.6
AE	33.5	0.1	5.7
RE	76.4	27.1	2.7
MBE	51.7	1.7	4.4
DE	55.7	5.0	4.1
ARE	77.4	30.8	2.6
MCE	83.8	35.4	2.1

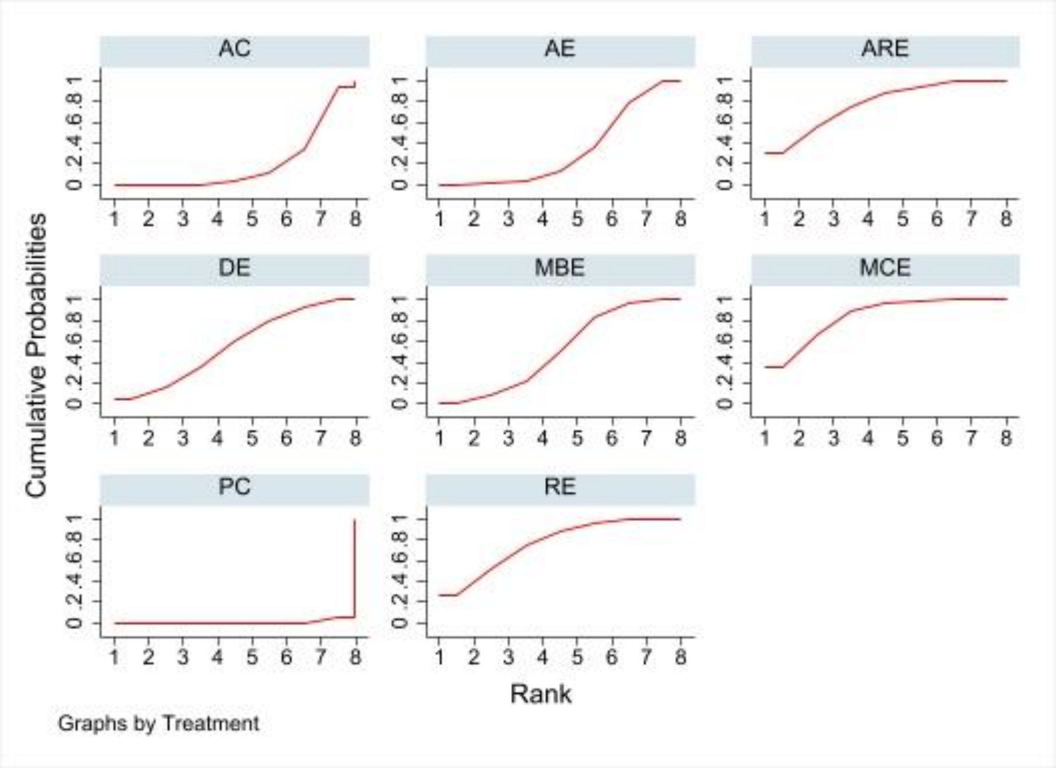

3. Analysis results of r=0.7

Treatm~t	SUCRA	PrBest	MeanRank
PC	0.6	0.0	8.0
AC	22.1	0.0	6.5
AE	32.8	0.1	5.7
RE	70.8	18.2	3.0
MBE	50.3	1.5	4.5
DE	58.1	6.4	3.9
ARE	79.2	32.7	2.5
MCE	86.0	41.0	2.0

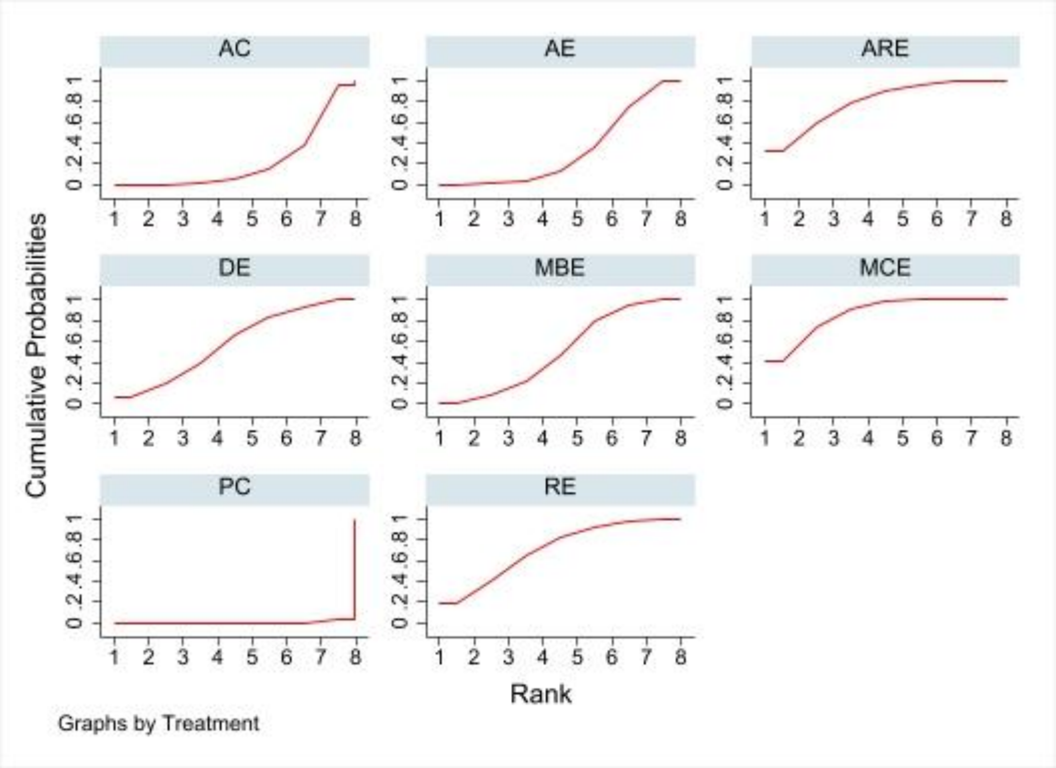

4. Analysis results of r=0.9

Treatm~t	SUCRA	PrBest	MeanRank
PC	1.2	0.0	7.9
AC	26.5	0.0	6.1
AE	32.8	0.2	5.7
RE	62.6	10.6	3.6
MBE	43.3	0.7	5.0
DE	68.2	14.2	3.2
ARE	79.9	34.7	2.4
MCE	85.5	39.6	2.0

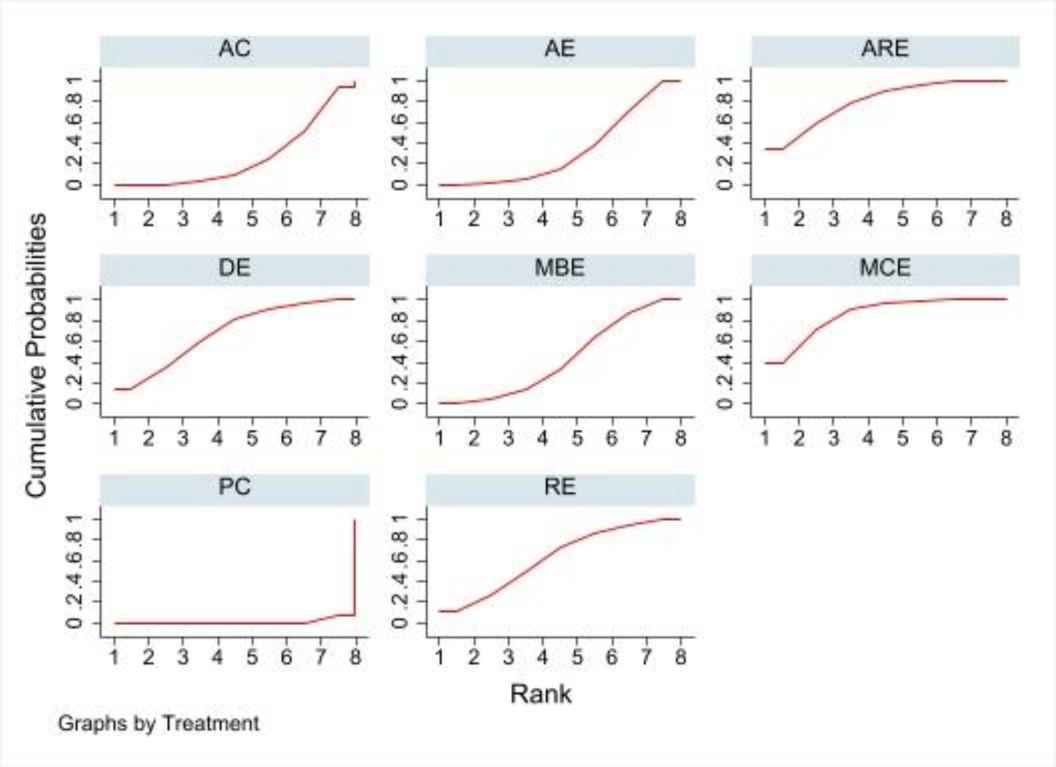
AC
AE
ARE
DE
MBE
MCE
PC
RE
Cumulative Probabilities
0 .2 .4 .6 .8 1
1 2 3 4 5 6 7 8
Rank
Graphs by Treatment
